# A novel series of pyrazole-platinum(II) complexes as potential anti-cancer agents that induce cell cycle arrest and apoptosis in breast cancer cells

**DOI:** 10.1080/14756366.2018.1471687

**Published:** 2018-06-04

**Authors:** Robert Czarnomysy, Arkadiusz Surażyński, Anna Muszynska, Agnieszka Gornowicz, Anna Bielawska, Krzysztof Bielawski

**Affiliations:** a Department of Synthesis and Technology of Drugs, Medical University of Bialystok, Bialystok, Poland;; b Department of Medicinal Chemistry, Medical University of Bialystok, Bialystok, Poland;; c Department of Biotechnology, Medical University of Bialystok, Bialystok, Poland

**Keywords:** Anti-cancer drugs, platinum complexes, pyrazole, apoptosis

## Abstract

Six novel compounds of platinum(II) with pyrazole derivatives PtPz1–PtPz6 were synthesised and characterised (PtPz1 - [Pt_2_N-hydroksymethyl-3,5-dimethylpyrazole_4_(berenil)_2_]Cl_4_; PtPz2 - [Pt_2_3,5-dimethylpyrazole_4_(berenil)_2_]Cl_4_; PtPz3 - [Pt_2_3,4-dimethylpyrazole_4_(berenil)_2_]Cl_4_; PtPz4 - [Pt_2_pyrazole_4_(berenil)_2_]Cl_4_; PtPz5- [Pt_2_5-methylpyrazole_4_(berenil)_2_]Cl_4_; PtPz6 - [Pt_2_N-ethylpyrazole_4_(berenil)_2_]Cl_4_). The cytotoxic activity of these complexes against MCF-7 and MDA-MB-231 breast cancer cell lines was determined using the MTT assay. Evaluation of apoptosis induction was done with the Annexin V-fluorescein isothiocyanate/propidium iodide assay. In addition, using a flow cytometer, we determined the influence of test compounds on the cell cycle and caspase-3, -8, and -9 activity. The obtained results of caspase activity were confirmed by cell imaging. Moreover, using the flow cytometer, the effects of the test compounds on mitochondrial potential change were assessed. The test results showed that novel pyrazole-platinum(II) complexes exhibited stronger anti-proliferative activity against two breast cancer cell lines than reference cisplatin. Compounds PtPz1, PtPz2, and PtPz3 with methyl substituents at the pyrazole ring showed stronger activity than pyrazole or ethylpyrazole containing complexes. Studies have shown that inhibition of cell survival occurs by arresting the G1 cell cycle and inducing apoptosis. Our analysis associated with the response of MCF-7 and MDA-MB-231 cells to treatment with PtPz1–PtPz6 showed that it leads the cells through the external and intrinsic (mitochondrial) apoptotic pathway via indirect DNA damage.

## Introduction

Breast cancer is one of the most commonly diagnosed malignancies leading to cancer-related deaths in women worldwide^1^. Although most conventional chemotherapies seem to result in success initially, they cannot eliminate the malignant clone totally and the disease relapses due to this therapy-resistant subpopulation^2^^,^^3^. Efforts at early detection and new therapeutic approaches to reduce mortality are used as a general treatment for breast cancer and other malignancies. Multiple mechanisms are involved in drug resistance, such as drug metabolism, membrane transporters, and apoptosis evasion^4^.

Since the discovery of cisplatin as one of the most successful anti-cancer drugs, thousands of novel platinum complexes have been synthesised and evaluated for their anti-cancer properties. Despite their sound results in cancer therapy, patients receiving platinum drugs may also experience severe side effects that subsequently limit their administration in clinical practice^5^. Therefore, in recent years many platinum-based compounds have been synthesised with the hope of eliminating the side effects of these drugs^6^. Results obtained from these studies indicated that Pt(II) complexes have significant anti-cancer activity comparable with cisplatin and cause a strong apoptotic response^6–10^. Our previous studies have shown that pyridine and dimethylpyridine platinum(II) complexes induce apoptosis via the mitochondrial pathway, with a decrease in mitochondrial membrane potential (MMP) and activation of caspase-9, as well as via the external pathway with a significant increase in FADD protein expression and caspase-8^8,9^. Additionally, dinuclear platinum(II) complexes increase the expression of NF-κB and decrease the expression of Akt, which leads to increased apoptosis^10^. Moreover, berenil-platinum(II) complexes bind to the minor groove of duplex DNA in A/T-rich regions, where they are thought to exert their biological activity through the inhibition of DNA-associated enzymes, such as DNA topoisomerases I and/or II, or possibly by direct transcription inhibition^11^^,^^12^.

In the case of breast cancer chemotherapy, the method to improve cisplatin selectivity may be combined treatment with cisplatin plus echistatin^13^. Likewise, monoclonal antibody against MUC1 increased the sensitivity of breast cancer cells to the dinuclear platinum(II) complex. The combined effects of a monoclonal antibody against MUC1 used together with a dinuclear platinum(II) complex showed high anti-proliferative properties and strong cytotoxic activity in MCF-7 and MDA-MB-231 breast cancer cell lines. This effect was much stronger than treatment with anti-MUC1, cisplatin, dinuclear platinum(II) complex, or cisplatin in the presence of anti-MUC1^14^.

**Figure 1.**
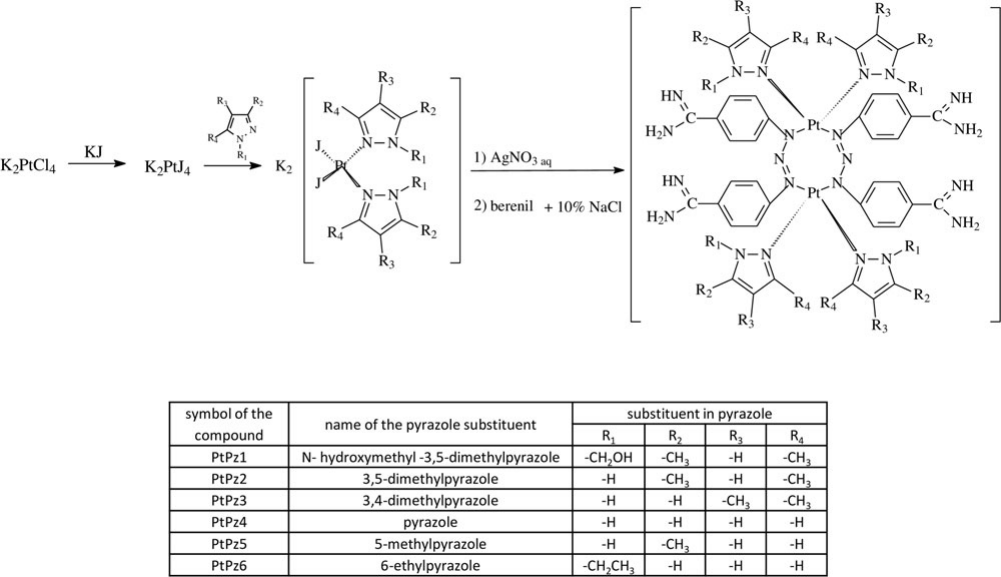
Synthesis scheme of PtPz1–PtPz6.

The goal of our work was to synthesise six novel pyrazole complexes of platinum(II) (Figure 1) and demonstrate that the synthesised compounds triggered a pro-apoptotic cascade in breast cancer cells. The advantage of pyrazole compounds is the ease of complexation of such metals as copper, ruthenium, palladium, or platinum, which strengthens their therapeutic effect. It can be concluded that the effect of ligands and corresponding metal-complexes depends not only on the specific features in the auxiliary ligand and metal ion, but also on cancer cell type. Moreover, results from the last studies suggest that both ancillary ligand and intercalative ligand influence the degree of binding of these complexes to DNA as a result of which the majority of the metal-pyrazole complexes possessed anti-proliferative activities against cancer cell lines^15–17^. Additionally, various pyrazole and pyrazoline derivatives have been identified as inhibitors of cyclin-dependent kinase^18^ and vascular endothelium growth factors^19^. Pyrazoloacridine was identified as a DNA topoisomerase inhibitor. It inhibited malignancy and induced apoptosis in myeloma and leukemia cells and displayed pre-clinical activity in myeloma and leukemia cells both *in vitro* and *in vivo*^20^. In view of the above, we developed a series of novel pyrazole-platinum(II) complexes that exhibited promising anti-tumor activity.

## Materials and methods

### Materials

Dimethylformamide, K_2_PtCl_4_, KI, acetone, N-hydroksymethyl-3,5-dimethylpyrazole, 3,5-dimethylpyrazole, 3,4-dimethylpyrazole, pyrazole, 5-methylpyrazole, N-ethylpyrazole, diethyl ether, methanol, ethidium bromide, cisplatin, 3–(4,5-dimethylthiazol-2-yl)-2,5-diphenyltetrazolium bromide (MTT), Triton X-100, formaldehyde, dimethylsulfoxide (DMSO) were purchased from Sigma Chemical Co. (St. Louis, MO, USA). Stock cultures of fibroblast cells, human MCF-7 breast cancer cells, and human MDA-MB-231 breast cancer cells were purchased from the American Type Culture Collection (ATCC, Manassas, VA). Dulbecco’s minimal essential medium (DMEM), fetal bovine serum (FBS), CPSR1, and PBS used in a cell culture were products of Gibco (San Diego, CA, USA). Glutamine, penicillin, and streptomycin were obtained from Quality Biologicals Inc. (Gaithersburg, MD, USA). Fluorescein isothiocyanate (FITC) Annexin V Apoptosis Detection Kit II, JC-1 MitoScreen Kit, APO-Direct Kit, anti-active-caspase-3 mouse monoclonal antibody, anti-active-caspase-8 mouse monoclonal antibody, anti-active-caspase-9 mouse monoclonal antibody, FITC anti-mouse secondary antibody were from BD Pharmingen (San Diego, CA); FLICA Caspase 3 Kit, FLICA Caspase 8 Kit, FLICA Caspase 9 Kit, Hoechst 33342 (ImmunoChemistry Technologies, Bloomington, MN, USA), RNase A Solution (Promega, Madison, WI, USA). All tested compounds were dissolved in DMSO.

### Physical measurements

The structure of a synthesised compound was confirmed by ^1^H-NMR and ^13^C-NMR spectra recorded on the Brucker AC 200F (Germany) apparatus (^1^H – 200 MHz and ^13^C – 50 MHz) in deuterated dimethylsulfoxide (d_6_-DMSO). Chemical shifts are expressed as a δ value (ppm). The multiplicity of resonance peaks is indicated as singlet (*s*), doublet (*d*), triplet (*t*), quartet (*q*), and multiplet (*m*). Infrared spectra were recorded on the Perkin-Elmer Spectrum 100 FT-IR spectrometer (PerSeptive Biosystems, Houston, TX, USA) as KBr pellets (4000–450 cm^−1^). Mass spectra were recorded using a Mariner mass spectrometer (USA). Melting points were determined on the Buchi 535 (GER) melting-point apparatus, and were uncorrected. Elemental analysis of C, H, and N was performed on a Perkin-Elmer 240 analyser (USA), and satisfactory results within ±0.4% of calculated values were obtained.

## Chemistry

### General preparation of platinum complexes (PtPz1–PtPz6)

K_2_PtCl_4_ (0.72 mmol) was dissolved in 40 ml of deionised water. KI (7.2 mmol) was added and the reaction mixture was stirred for 30 min. Then, the corresponding pyrazole derivative (N-hydroxymethyl-3,5-dimethylpyrazole; 3,5-dimethylpyrazole; 3,4-dimethylpyrazole; pyrazole; 5-methylpyrazole or *N*-ethylpyrazole (1.44 mmol)) was added to the reaction mixture while stirring, to obtain a precipitate. The stirring was continued for 24 h and the precipitate was then collected by filtration. This compound was filtered, then was washed with 30 ml deionised water and dried in a vacuum. A suitable derivative (cis-[Pt(N-hydroxymethyl-3,5-dimethylpyrazole)_2_I_2_]; cis-[Pt(3,5-dimethylpyrazole)_2_I_2_]; cis-[Pt(3,4-dimethylpyrazole)_2_I_2_]; cis-[Pt(pyrazole)_2_I_2_]; cis-[Pt(5-methylpyrazole)_2_I_2_]; or cis-[Pt(N-ethylpyrazole)_2_I_2_] (1.22 mmol)) was suspended in 5 ml of an aqueous solution of silver nitrate (AgNO_3_) (2.44 mmol). The reaction mixture was stirred for 24 h at room temperature in the dark. The AgI precipitate was filtered off. Berenil (1.22 mmol) and a solution of 10% NaCl (5 ml) were added to the filtrate and stirred until a precipitate of the corresponding pyrazole derivative platinum complex formed. Afterwards, the product was filtered off and washed with a small amount of diluted HCl, deionised water, methanol, acetone, and ethyl ether, and dried under vacuum.

*[Pt_2_(N-hydroxymethyl-3,5-dimethylpyrazole)_4_(berenil)_2_]·4HCl·2H_2_O (****PtPz1****):* Yield: 62.4%; yellow powder; mp 238–240 °C; ^1^H-NMR (DMSO-d_6_) δ (ppm): 9.24 (br, s, amidine), 7.92 (d, *J* = 8.6 Hz, 8H, Ar), 7.68 (d, *J* = 8.3 Hz, 8H, Ar), 6.15 (s, 4H, Pz), 5.95 (s, 8H, CH_2_), 4.15 (s, 4H, OH), 2.40 (s, 12H, CH_3_), 2.30 (s, 12H, CH_3_); ^13^C-NMR (DMSO-d_6_) δ (ppm): 165.2 (amidine), 149.5 (Ar), 147.2 (Pz), 140.7 (Pz), 129.5 (Ar), 122.0 (Ar), 118.0 (Ar), 105.8 (Pz), 73.8 (CH_2_), 13.6 (CH_3_), 11.0 (CH_3_); IR (KBr, cm^−1^): 3213 (C=NH imine/OH alcohol), 3143 (NH_3_^+^), 2925 (CH_2_/CH_3_), 1687 (NCN/C=N imine), 1607 (triazene), 1572 (CN pyrazole ring), 1514 (NH_3_^+^), 1485 (CH_2_/CH_3_), 1440 (C-O alcohol), 1259 (triazene), 1175 (triazene), 1041 (C-O alcohol), 800 (1,4-disubstituted aromatic), 599 (Pt-N); MS (ES, HR) *m/z* (M^+^) calcd. for C_52_H_72_Cl_4_N_22_O_4_Pt_2_ 1601.2660, found 1601.2689; Anal. calcd. for C_52_H_68_N_22_O_4_Pt_2_·4HCl·2H_2_O: C, 38.24; H, 4.44; N, 18.87; found: C, 38.27; H, 4.46 N, 18.86.

*[Pt_2_(3,5-dimethylpyrazole)_4_(berenil)_2_]·4HCl·2H_2_O (****PtPz2****):* Yield: 77.6%; yellow powder; mp 254–257 °C; ^1^H-NMR (DMSO-d_6_) δ (ppm): 12.10 (br, s, NH), 9.24 (br, s, amidine), 7.92 (d, *J* = 8.6 Hz, 8H, Ar), 7.68 (d, *J* = 8.3 Hz, 8H, Ar), 5.73 (s, 4H, Pz), 2.14 (s, 24H, CH_3_); ^13^C-NMR (DMSO-d_6_) δ (ppm): 165.2 (amidine), 149.5 (Ar), 143.2 (Pz), 129.5 (Ar), 122.0 (Ar), 118.0 (Ar), 103.4 (Pz), 13.2 (CH_3_), 13.4 (CH_3_); IR (KBr, cm^−1^): 3238 (C=NH imine), 3143 (NH_3_^+^), 2923 (CH_3_), 1686 (NCN/C=N imine), 1606 (triazene), 1572 (CN pyrazole ring), 1513 (NH_3_^+^), 1483 (CH_3_), 1257 (triazene), 1174 (triazene), 855 (1,4-disubstituted aromatic), 525 (Pt-N); MS (ES, HR) *m/z* (M^+^) calcd. for C_48_H_64_Cl_4_N_22_Pt_2_ 1481.1620, found 1481.1600; Anal. calcd. for C_48_H_60_N_22_Pt_2_·4HCl·2H_2_O: C, 38.00; H, 4.52; N, 20.31; found: C, 38.01; H, 4.54 N, 20.27.

*[Pt_2_(3,4-dimethylpyrazole)_4_(berenil)_2_]·4HCl·2H_2_O (****PtPz3****):* Yield: 60.4%; yellow powder; mp 227–229 °C; ^1^H-NMR (DMSO-d_6_) δ (ppm): 12.52 (br, s, NH), 9.24 (br, s, amidine), 7.92 (d, *J* = 8.6 Hz, 8H, Ar), 7.68 (d, *J* = 8.3 Hz, 8H, Ar), 7.45 (s, 4H, Pz), 2.12 (s, 12H, CH_3_), 1.94 (s, 12H, CH_3_); ^13^C-NMR (DMSO-d_6_) δ (ppm): 165.2 (amidine), 149.5 (Ar), 140.3 (Pz), 134.8 (Pz), 129.5 (Ar), 122.0 (Ar), 118.0 (Ar), 111.6 (Pz), 12.3 (CH_3_), 7.9 (CH_3_); IR (KBr, cm^−1^): 3218 (C=NH imine), 3134 (NH_3_^+^), 2919 (CH_3_), 1686 (NCN/C=N imine), 1606 (triazene), 1528 (CN pyrazole ring), 1513 (NH_3_^+^), 1439 (CH_3_), 1257 (triazene), 1174 (triazene), 854 (1,4-disubstituted aromatic), 525 (Pt-N); MS (ES, HR) *m/z* (M^+^) calcd. for C_48_H_64_Cl_4_N_22_Pt_2_ 1481.1620, found 1481.1620; Anal. calcd. for C_48_H_60_N_22_Pt_2_·4HCl·2H_2_O: C, 38.00; H, 4.52; N, 20.31; found: C, 38.02; H, 4.56 N, 20.32.

*[Pt_2_(pyrazole)_4_(berenil)_2_]·4HCl·2H_2_O (****PtPz4****):* Yield: 29.7%; lemon powder; mp 243–245 °C; ^1^H-NMR (DMSO-d_6_) δ (ppm): 11.84 (br, s, NH), 9.48 (br, s, amidine), 7.92 (d, *J* = 8.6 Hz, 8H, Ar), 7.67 (d, *J* = 8.3 Hz, 8H, Ar), 7.65 (d, *J* = 2.0 Hz, 4H, Pz), 6.40 (t, *J* = 2.6 Hz, 4H, Pz); ^13^C-NMR (DMSO-d_6_) δ (ppm): 165.2 (amidine), 153.8 (Ar), 146.3 (Pz), 140.8 (Pz), 130.2 (Ar), 121.6 (Ar), 114.6 (Ar), 107.0 (Pz); IR (KBr, cm^−1^): 3220 (C=NH imine), 3127 (NH_3_^+^), 1686 (NCN/C=N imine), 1606 (triazene), 1567 (CN pyrazole ring), 1513 (NH_3_^+^), 1257 (triazene), 1174 (triazene), 854 (1,4-disubstituted aromatic), 525 (Pt-N); MS (ES, HR) *m/z* (M^+^) calcd. for C_40_H_48_Cl_4_N_22_Pt_2_ 1366.2482, found 1366.2503; Anal. calcd. for C_40_H_44_N_22_Pt_2_·4HCl·2H_2_O: C, 34.20; H, 3.73; N, 21.93; found: C, 34.18; H, 3.76 N, 21.92.

*[Pt_2_(5-methylpyrazole)_4_(berenil)_2_]·4HCl·2H_2_O (****PtPz5****):* Yield: 38.6%; yellow powder; mp 218–221°C; ^1^H-NMR (DMSO-d_6_) δ (ppm): 12.43 (br, s, NH), 9.48 (br, s, amidine), 7.92 (d, *J* = 8.6 Hz, 8H, Ar), 7.67 (d, *J* = 8.3 Hz, 8H, Ar), 7.48 (d, *J* = 2.8 Hz, 4H, Pz), 6.12 (d, *J* = 2.8 Hz, 4H, Pz), 2.28 (s, 12H, CH_3_); ^13^C-NMR (DMSO-d_6_) δ (ppm): 165.2 (amidine), 153.8 (Ar), 139.4 (Pz), 137.6 (Pz), 130.2 (Ar), 121.6 (Ar), 114.6 (Ar), 103.2 (Pz), 13.2 (Pz); IR (KBr, cm^−1^): 3208 (C=NH imine), 3135 (NH_3_^+^), 1686 (NCN/C=N imine), 1606 (triazene), 1570 (CN pyrazole ring), 1513 (NH_3_^+^), 1439 (CH_3_), 1257 (triazene), 1173 (triazene), 851 (1,4-disubstituted aromatic), 525 (Pt-N); MS (ES, HR) *m/z* (M^+^) calcd. for C_44_H_56_Cl_4_N_22_Pt_2_ 1425.0540, found 1425.0620; Anal. calcd. for C_44_H_52_N_22_Pt_2_·4HCl·2H_2_O: C, 36.17; H, 4.14; N, 21.09;, found: C, 36.19; H, 4.13 N, 21.11.

*[Pt_2_(N-ethylpyrazole)_4_(berenil)_2_]·4HCl·2H_2_O (****PtPz6****):* Yield: 69.9%; lemon powder; mp 255–260 °C; ^1^H-NMR (DMSO-d_6_) δ (ppm): 9.48 (br, s, amidine), 7.92 (d, *J* = 8.6 Hz, 8H, Ar), 7.67 (d, *J* = 8.3 Hz, 8H, Ar), 7.83 (d, *J* = 2.0 Hz, 4H, Pz), 7.27 (d, *J* = 7.5 Hz, 4H, Pz), 6.23 (t, *J* = 2.6 Hz, 4H, Pz), 4.10 (q, *J* = 7.2 Hz, 8H, CH_2_), 1.46 (t, *J* = 7.2 Hz, 12H, CH_2_); ^13^C-NMR (DMSO-d_6_) δ (ppm): 165.2 (amidine), 153.8 (Ar), 139.6 (Pz), 130.2 (Ar), 129.7 (Pz), 121.6 (Ar), 114.6 (Ar), 105.3 (Pz), 48.3 (CH_2_), 15.7 (CH_3_); IR (KBr, cm^−1^): 3360 (C=NH imine), 3109 (NH_3_^+^), 2924 (CH_2_/CH_3_), 1686 (NCN/C=N imine), 1606 (CN pyridine/triazene), 1572 (CN pyrazole ring), 1514 (NH_3_^+^), 1485 (CH_2_/CH_3_), 1257 (triazene), 1173 (triazene), 851 (1,4-disubstituted aromatic), 525 (Pt-N); MS (ES, HR) *m/z* (M^+^) calcd. for C_48_H_64_Cl_4_N_22_Pt_2_ 1481.1620, found 1481.1820; Anal. calcd. for C_48_H_60_N_22_Pt_2_·4HCl·2H_2_O: C, 38.00; H, 4.52; N, 20.31, found: C, 37.99; H, 4.53 N, 20.36.

## Biological activity

### Cell lines and cell culture

MCF-7, MDA-MB-231 (both human breast cancer cell lines), and fibroblast cells were obtained from American Type Culture Collection (ATCC, Manassas, VA, USA). DMEM and FBS used in a cell culture were from Gibco (USA). Glutamine, penicillin, and streptomycin were obtained from Quality Biologicals Inc. (USA). DMEM media was blended with 50 units/ml of penicillin, 50 μg/ml of streptomycin, 10% of FBS. All cell lines were cultured in 5% CO_2_ and fully humidified at 37 °C. Cells were cultured in Costar flasks and sub-confluent cells were detached with 0.05% trypsin and 0.02% ethylenediaminetetraacetic acid in calcium-free phosphate-buffered saline (PBS), counted in hemocytometers, and plated at 5 × 10^5^ cells/well of six-well plates (Thermo Scientific, New York, NY, USA) in 2 ml of growth medium (DMEM without phenol red with 10% CPSR1). Cells reached about 80% of confluency at day 2, and in most cases such cells were used for the assays.

### Cell viability assay

The viability of cultured cells was decided through assaying the reduction of MTT to formazan. In brief, MCF-7, MDA-MB-231, and fibroblast cells line were seeded at an initial density of 1 × 10^5^ cells per well. Then, the cells were incubated at 37 °C for 24 h. Subsequently, cultured cells were treated with a medium containing concentrations (5, 10, 20, 30, 40, and 50 μM) of PtPz1–PtPz6 for 24 h and 48 h. After the incubation period, MTT was added into all wells in the final concentration of 0.5 mg/ml. After that, the cells were incubated at 37 °C for 4 h. Then, by removing the medium, 200 μl of DMSO was added to all wells. As a result, insoluble formazan was dissolved in DMSO (0.5%). At 570 nm (630 nm as a reference), the absorbance was measured in an Evolution 201 reader (Thermo Scientific, Waltham, MA).

### Cell morphological analysis

To visualise their morphological specificity, the MCF-7 and MDA-MB-231 cells were exposed to PtPz1–PtPz6 treatment. The cells, at a density of 2.5 × 10^5^, were seeded into six-well plates and incubated with the tested complexes (20 μM) at 37 °C in a humidified atmosphere containing 5% CO_2_ for 24 h. After incubation, the cells were washed with PBS two times. The cells were visualised using a phase contrast microscope (Nikon Eclipse Ti, Japan) at a 100× magnification.

### Cell cycle analysis

Distribution of cell cycle phases was analysed by flow cytometry. Briefly, MCF-7 and MDA-MB-231 cells were seeded into six-well plates at a density of 2.5 × 10^5^ cells per well and treated with 20 μM of PtPz1–PtPz6 for 24 h. After incubation, the cells were harvested and then fixed with 1 ml of 70% ethanol and kept overnight at −20 °C. Before analysis, the cells were re-suspended in PBS, treated with 50 μg/ml of DNase-free RNase A Solution (Promega), and stained with 100 μg/ml of PI. The FACSCanto II flow cytometer (BD Biosciences Systems, San Jose, CA) was used to read the fluorescence.

### Analysis of cyclin D1

**Cell lysates**: Briefly, trypsinised cells were washed three times with cold PBS and centrifuged at 1000 *g* for 5 min at 4 °C. The cells (1 × 10^6^) were suspended in lysis buffer for whole cell lysates. After centrifugation, the supernatants were frozen immediately at −70 °C. The concentration of human G1/S-specific cyclin D1 was measured. Cells without addition of compounds were treated as controls.

**Determination of human G1/S-specific cyclin D1**: The high sensitivity assay kit (EIAab) was used to determine the concentration of human G1/S-specific cyclin D1 in cell lysates. The microtiter plate provided in this kit has been pre-coated with an antibody specific to cyclin D1. Standards and samples were added to the appropriate microtiter plate wells. After 2 h of incubation at 37 °C, the plate was incubated with biotin-conjugated antibody for 1 h at 37 °C. Then microplate wells were aspirated and washed three times and then incubated with avidin conjugated to horseradish peroxidase (HRP). Then, a TMB substrate solution was added to each well. Those wells that contained target antigen exhibited a change in color. The enzyme–substrate reaction was terminated by the addition of a sulfuric acid solution and the color change was measured spectrophotometrically at a wavelength of 450 nm ± 2 nm. The concentration of cyclin D1 in the samples was determined by comparing the O.D. of the samples to the standard curve.

### Flow cytometry assessment of Annexin V binding

To characterise the mode of cell death induced by PtPz1–PtPz6, a flow cytometry analysis was performed using Apoptosis Detection Kit II (BD Pharmingen, San Diego, CA), according to the manufacturer’s instructions. Cells (10000 cells measured) were analysed in a flow cytometer (BD FACSCanto II flow cytometer, San Jose, CA). Annexin V bound with high affinity to phosphatidylserine and thus could be used to identify cells in all stages of the programmed cell death. Propidium iodide (PI) stained cells with a disrupted cell membrane, and it could be used to identify late apoptotic and dead cells^13^. Cells cultured in a drug-free medium were used as controls. Optimal parameter settings were found using a positive control (cells incubated with 3% formaldehyde in buffer during 30 min on ice). Results were analysed with FACSDiva software (BD Biosciences Systems, San Jose, CA).

### DNA fragmentation assay

DNA fragmentation associated with apoptosis was examined by the terminal deoxynucleotidyltransferase (TdT)- mediated dUTP nick end labeling (TUNEL) method using a commercial assay kit (APO-Direct Kit; BD Pharmingen, San Diego, CA). After treatment, the cells were fixed with 1% paraformaldehyde in PBS (4°C, 30 min), washed in PBS, and permeabilised with ice-cold 70% ethanol. The APO-Direct Kit TUNEL assay was performed as described by the manufacturer. Briefly, the fixed cells were washed twice using the kit wash buffer, and after centrifugation the supernatant was discarded. The DNA labeling solution (containing TdT enzyme and FITC-dUTP) was added to the cell pellet, and the re-suspended mixture was incubated for 1 h at 37°C with occasional shaking. At the end of incubation time, rinse buffer was added, and after centrifugation the supernatant was discarded. We repeated the cell rinsing with rinse buffer, and then suspended the cell pellet in PI/RNase staining buffer. Cells were incubated at room temperature for 30 min and immediately analysed in a BD FACSCanto II flow cytometer. In total, 10,000 events were collected per test sample. The results were analysed with FACSDiva software (both from BD Biosciences Systems, San Jose, CA). The percentage of cells with distinctive apoptotic DNA strand breaks and distinguished by green fluorescent emission was calculated.

### Analysis of mitochondrial membrane potential

Disruption of the MMP was assessed using the lipophilic cationic probe 5,5′,6,6′-tetrachloro-1,1′,3,3′-tetraethylbenzimidazolcarbocyanine iodide (JC-1 MitoScreen kit; BD Biosciences Systems, San Jose, CA), as described previously^9^. Briefly, unfixed cells were washed and re-suspended in PBS supplemented with 10 µg/ml of JC-1. Cells were then incubated for 15 min at room temperature in the dark, washed, and re-suspended in PBS for immediate BD FACSCanto II flow cytometry analysis. The percentage of cells with disrupted MMP was calculated using FACSDiva software (both from BD Biosciences Systems, San Jose, CA).

### Caspase-3 enzymatic activity assay

Caspase-3 activity was measured using the FLICA Caspase 3 Assay Kit (ImmunoChemistry Technologies, Bloomington, MN, USA), according to the manufacturer’s instructions. Briefly, cultured MDA-MB-231 breast cancer cells (1 × 10^6^) were washed with cold PBS twice and re-suspended in buffer. We added 5 µl of diluted FLICA reagent and 2 µl of Hoechst 33342 to 93 µl of cell suspension and mixed by pipetting. The cells were incubated for 60 min at 37°C. After incubation, the cells were washed twice in 400 µl of apoptosis wash buffer and centrifuged at 300 × *g*. After the last wash, we re-suspended the cells in 100 µl of apoptosis wash buffer supplemented with 10 µg/ml PI. Analysis was performed using the BD FACSCanto II flow cytometer, and the results were analysed with FACSDiva software (both from BD Biosciences Systems, San Jose, CA).

### Caspase-8 enzymatic activity assay

Caspase-8 activity was measured using the FLICA Caspase 8 Assay Kit (ImmunoChemistry Technologies, Bloomington, MN, USA), according to the manufacturer’s instructions. Briefly, cultured MCF-7 and MDA-MB-231 breast cancer cells (1 × 10^6^) were washed with cold PBS twice and re-suspended in buffer. We added 5 µl of diluted FLICA reagent and 2 µl of Hoechst 33342 to 93 µl of cell suspension and mixed by pipetting. The cells were incubated for 60 min at 37°C. After incubation, the cells were washed twice in 400 µl of apoptosis wash buffer and centrifuged at 300 × *g*. After the last wash, we re-suspended the cells in 100 µl of apoptosis wash buffer supplemented with 10 µg/ml PI. Analysis was performed using the BD FACSCanto II flow cytometer, and the results were analysed with FACSDiva software (both from BD Biosciences Systems, San Jose, CA).

### Caspase-9 enzymatic activity assay

Caspase-9 activity was measured using the FLICA Caspase 9 Assay Kit (ImmunoChemistry Technologies, Bloomington, MN, USA), according to the manufacturer’s instructions. Briefly, cultured MCF-7 and MDA-MB-231 breast cancer cells (1 × 10^6^) were washed with cold PBS twice and re-suspended in buffer. We added 5 µl of diluted FLICA reagent and 2 µl of Hoechst 33342 to 93 µl of cell suspension and mixed by pipetting. The cells were incubated for 60 min at 37°C. After incubation, the cells were washed twice in 400 µl of apoptosis wash buffer and centrifuged at 300 × *g*. After the last wash, we re-suspended cells in 100 µl of apoptosis wash buffer supplemented with 10 µg/ml PI. Analysis was performed using the BD FACSCanto II flow cytometer, and the results were analysed with FACSDiva software (both from BD Biosciences Systems, San Jose, CA).

### Fluorescence microscopy

Cells were seeded in BD Falcon™ 96-well black/clear bottom tissue culture plates optimised for imaging applications at 10,000 cells per well. After 24 h incubation with the tested compounds, the cells were rinsed with PBS and fixed with 3.7% formaldehyde solution in PBS for 10 min, washed three times with PBS, and permeabilised in 0.1% Triton X-100 solution for 5 min. After washing with PBS twice, non-specific binding was blocked by adding 3% FBS solution for 30 min. After that time, the cells were rinsed, incubated with anti-caspase-3 mouse monoclonal antibody, anti-caspase-8 mouse monoclonal antibody, anti-caspase-9 mouse monoclonal antibody (BD Pharmingen, San Diego, CA; 1:500) for 1 h, washed three times with PBS, and incubated with FITC anti-mouse secondary antibody (BD Pharmingen, San Diego, CA; 1:1000) in the dark for 60 min. After washing, the nuclei were stained with Hoechst 33342 and analysed with a fluorescent microscope (BD Pathway 855 confocal system) using a 40 × (0.75 NA) objective. Cell populations were analysed for cytoplasmic/nuclear fluorescence intensity. Images of FITC-labeled cells were acquired using a 488/10 excitation laser and a 515LP emission laser. Samples were visualised with a confocal laser scanning microscope (BD Pathway 855 Bioimager) using AttoVision software.

### Statistical analysis

All numerical data are presented as mean ± standard deviation (SD) from at least three independent experiments. Statistical analysis was conducted using the Origin 7.5 software (OriginLab, Northampton, MA, USA). Statistical differences in multiple groups were determined by one-way ANOVA followed by Tukey’s test. *p* < .05 was considered statistically significant.

## Results

### Chemistry section

Pyrazole-platinum (II) complexes (PtPz1–PtPz6) were obtained by two-step synthesis (Figure 1). In the first step, K_2_PtCl_4_ was treated with the appropriate substituted pyrazole in the presence of KI. After isolation and purification, the obtained intermediates were used for the second stage. For this purpose, the previously obtained compounds were treated with an aqueous solution of AgNO_3_ followed by berenil in a 10% NaCl. Since it is known that berenil, having functional and triazene groups, may bind to metals as linear monodentate, chelating bidentate, and in bridging bidentate modes, we have performed IR, ^1^H-, and ^13^C-NMR spectra of the synthesised compounds to determine the mode of metal-berenil bonding. The spectra show that binding of the metal to the amidino-group of the ligand does not occur since the frequencies attributed to the amidino-moiety, v(CN) = 1686–1687 cm^−1^ (for free berenil 1668 cm^−1^) as well as the signal in ^13^C-NMR of the carbon from the amidine group remain unaltered relative to the free ligand, 165.2 ppm. The presence of two broad singlets between 9.24 and 9.48 ppm attributed to the protonated residue in ^1^H-NMR indicate, moreover, the absence of this type of metal bonding. The presence of three bands at 1606–1607, 1257–1259, 1173–1175 cm^−1^ in the IR spectrum shows, on the other hand, that the triazene group coordinates in linear or bridging modes. The IH- and ^13^C-NMR spectra further support this mode of binding since the signal corresponding to the AA′BB′ system of the nearest protons of the triazene group are now broadened and distorted. The displacement at a low field 0.1 ppm for PtPz1–PtPz3 and 4.4 ppm for PtPz4–PtPz6 and the displacement at a high field 2.8 ppm for PtPz1–PtPz3 and 3.2 ppm for PtPz4-PtPz6 in ^13^C-NMR spectra (relative to the spectrum of free berenil) indicates, moreover, that the berenil ligand binds to Pt through the triazene group. Thus, the spectra and the micronalytical data, together with the spectrum of PtPz1–PtPz6, suggest that the compounds are a dimers where Pt binds to the nitrogen atoms of two triazene groups in a bridging mode.

### Cytotoxicity assay

**Figure 2.**
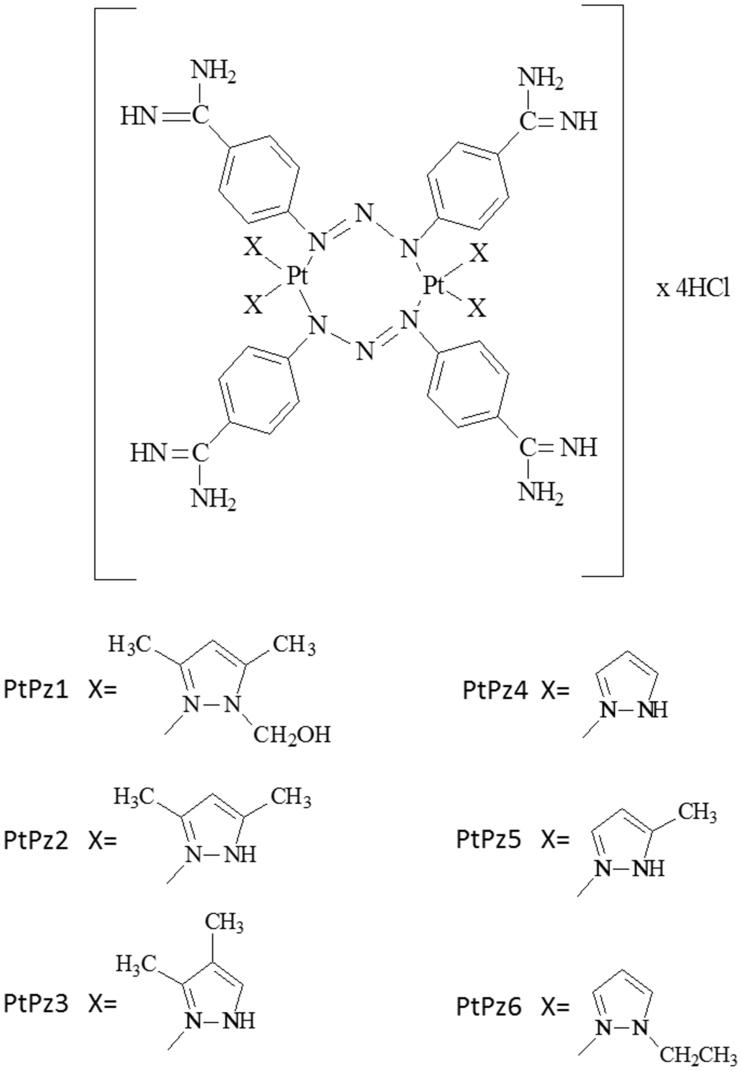
Chemical structures of PtPz1–PtPz6.

**Figure 3.**
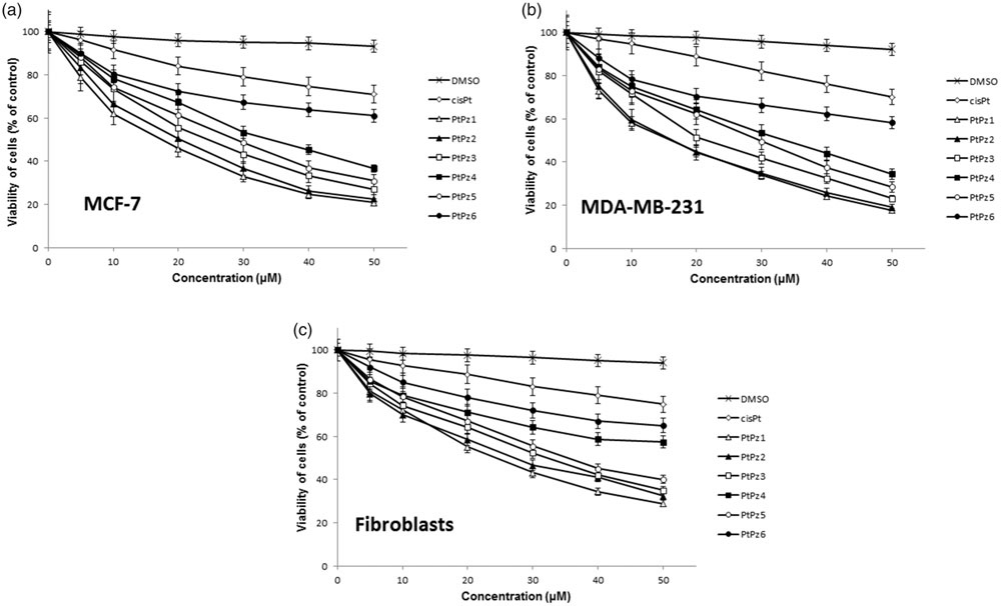
Viability of MCF-7 breast cancer cells (a), MDA-MB-231 breast cancer cells (b), and fibroblast cells (c) treated for 24 h with different concentrations of the tested compounds (PtPz1–PtPz6). Mean ± SD values from three independent experiments (*n* = 3) done in duplicate are presented.

**Figure 4.**
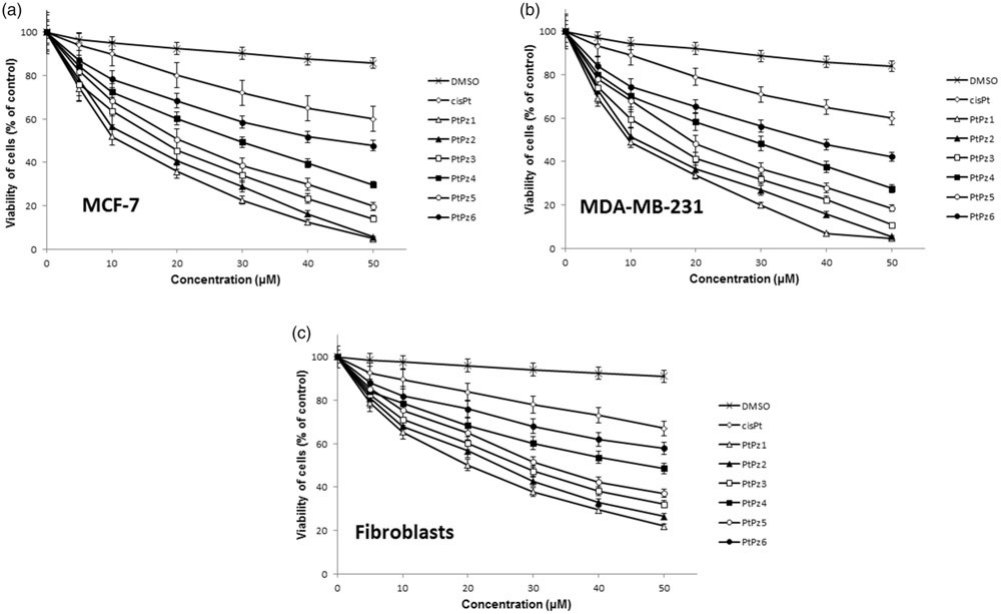
Viability of MCF-7 breast cancer cells (a), MDA-MB-231 breast cancer cells (b), and fibroblast cells (c) treated for 48 h with different concentrations of the tested compounds (PtPz1–PtPz6). Mean ± SD values from three independent experiments (*n* = 3) done in duplicate are presented.

To determine the effects of PtPz1–PtPz6 (Figure 2) compounds on the viability of breast cancer MCF-7, MDA-MB-231, and fibroblast cells, MTT assays were performed. The tested cells were treated with PtPz1–PtPz6 at increasing concentrations for 24 h (Figure 3) and 48 h (Figure 4). The study has shown that increasing the concentration of novel pyrazole-platinum(II) complexes decreases cell viability of the examined cells and thus contributes to greater cytotoxicity of the test compounds. In MCF-7 cells, IC_50_ values for PtPz1 were equal to 17 ± 2 μM, PtPz2 20 ± 2 μM, PtPz3 23 ± 1 μM, PtPz4 32 ± 2 μM, PtPz5 28 ± 2 μM, and PtPz6 above 50 μM after 24 h incubation; and PtPz1 11 ± 1 μM, PtPz2 13 ± 2 μM, PtPz3 18 ± 1 μM, PtPz4 29 ± 2 μM, PtPz5 22 ± 2 μM, and PtPz6 48 ± 2 μM after 48 h incubation. In MDA-MB-231 cells (24 h incubation), IC_50_ values were of 16 ± 1 μM for PtPz1, 17 ± 2 μM for PtPz2, 20 ± 2 μM for PtPz3, 33 ± 3 μM for PtPz4, 29 ± 1 μM for PtPz5, and above 50 μM for PtPz6. In case of 48 h incubation time IC_50_ values were of 10 ± 1 μM for PtPz1, 11 ± 1 μM for PtPz2, 16 ± 1 μM for PtPz3, 29 ± 2 μM for PtPz4, 19 ± 2 μM for PtPz5, and 39 ± 2 μM for PtPz6. After 24 h and 48 h of incubation, IC_50_ values for cisplatin in MCF-7 and MDA-MB-231 cells were above 50 μM. Although PtPz1–PtPz6 reduced the viability of breast cancer cells in a concentration-dependent manner, the viability of fibroblast cells was higher than the breast cancer cells after exposure to PtPz1–PtPz6. The IC_50_ values for fibroblasts after 24 h incubation were: PtPz1 24 ± 3 μM, PtPz2 27 ± 2 μM, PtPz3 32 ± 1 μM, PtPz4 above 50 μM, PtPz5 35 ± 1 μM, and PtPz6 above 50 μM; after 48 incubation PtPz1 19 ± 2 μM, PtPz2 24 ± 2 μM, PtPz3 28 ± 1 μM, PtPz4 46 ± 2 μM, PtPz5 31 ± 2 μM, PtPz6 above 50 μM and cisplatin above 50 μM, respectively. Due to the fact that at 50 μM cell viability for cisplatin on the three cell lines may seem quite similar, additional test was performed for this compound in concentrations allowing to determine IC_50_ values. After 24 h of incubation, IC_50_ values for cisplatin in MCF-7, MDA-MB-231, and fibroblast cells were 93 ± 2 μM, 82 ± 2 μM, above 100 μM, and in case 48 h of incubation: 78 ± 1 μM, 72 ± 1 μM, and above 100 μM, respectively. All IC_50_ values are shown in the supplementary material.

The obtained results suggest that breast cancer cells were more sensitive to PtPz1–PtPz6 than normal cells.

### The effect of PtPz1–PtPz6 on cell morphology

**Figure 5.**
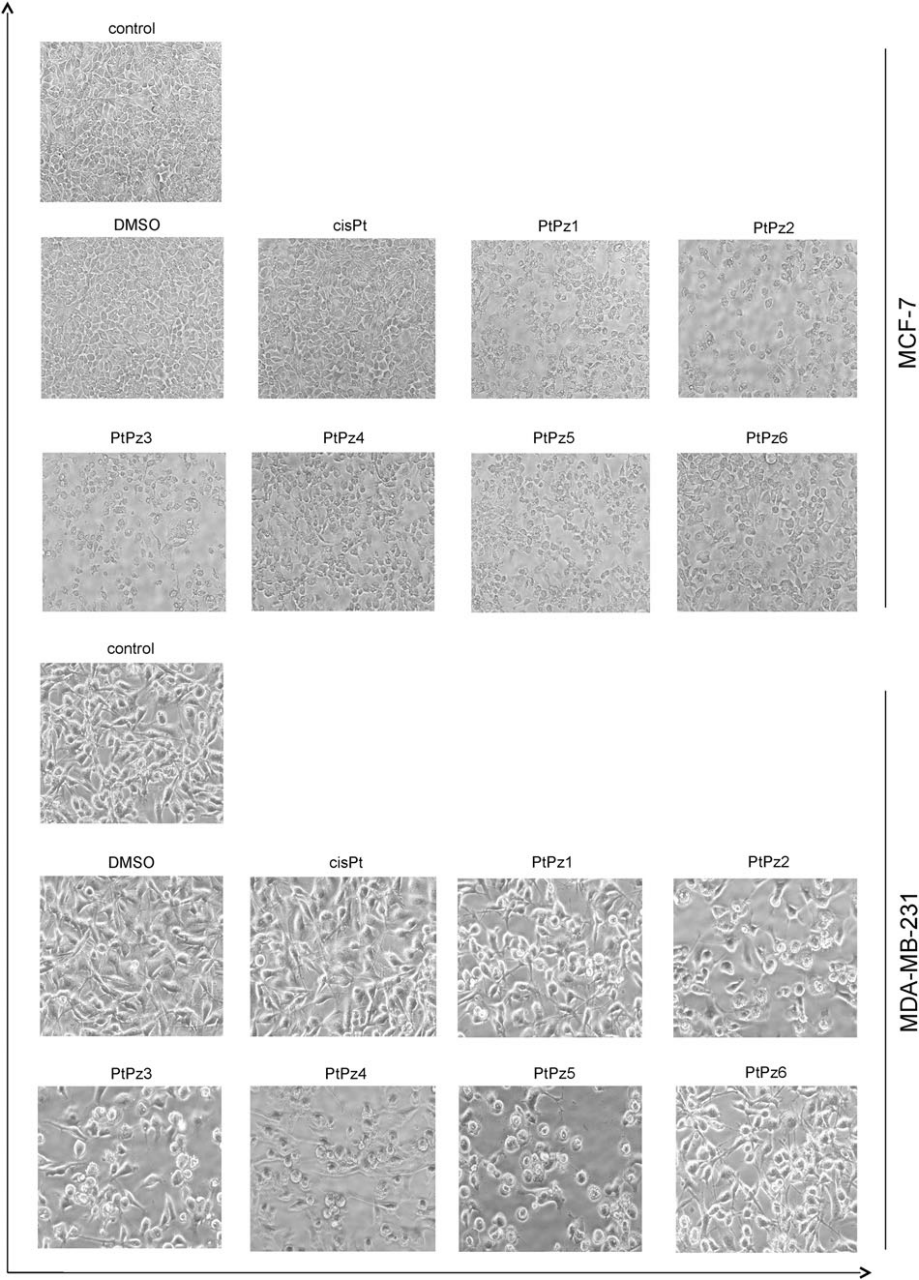
Morphological changes in MCF-7 and MDA-MB-231 cells incubated with 20 µM of PtPz1–PtPz6 for 24 h exposure. Representative photographs are shown. Morphological effects evaluated by phase contrast microscopy (magnification ×100).

Microscopic observations were carried out to see if the diminished cell viability of MCF-7 and MDA-MB-231 cells was accompanied by alterations in cell morphology and increased cell death. Figure 5 presents an overview of MCF-7 and MDA-MB-231 cells observed under phase contrast microscope after 24 h of incubation with the tested compounds in a concentration of 20 μM. Observations showed that PtPz1–PtPz6 stimulated cells had different cell shapes and cell densities. The densities of both cell lines were visibly reduced. The most significant suppression of cell proliferation was observed in cultures treated with PtPz1, PtPz2, and PtPz3. These results are in accord with those obtained in the cytotoxicity assay.

### Alteration of MCF-7 and MDA-MB-231 cell cycle progression by PtPz1–PtPz6

**Figure 6.**
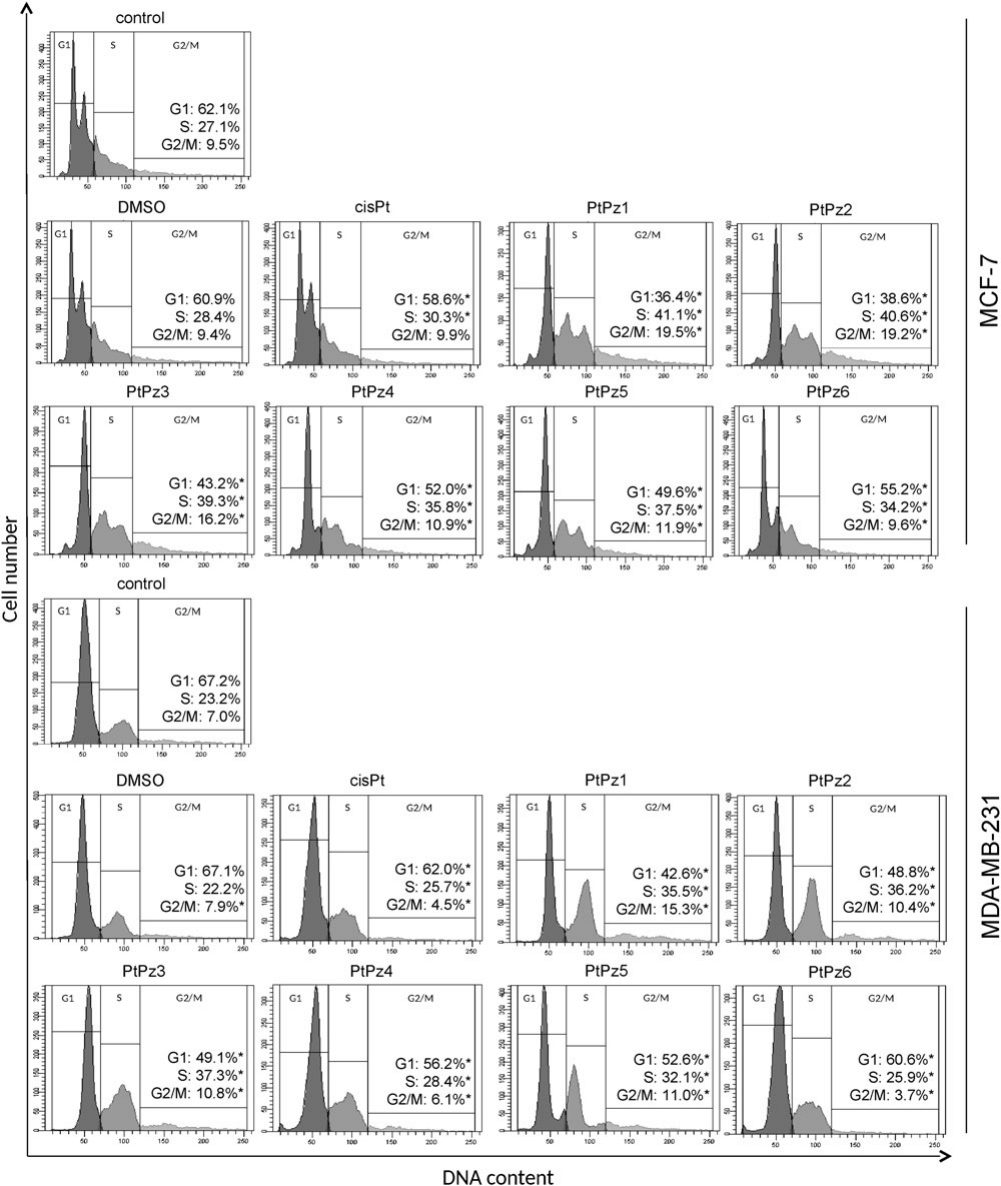
Flow cytometric analysis of cell cycle of MCF-7 and MDA-MB-231 breast cancer cells after 24 h of incubation with PtPz1–PtPz6 (20 µM) and cisplatin (20 µM) using propidium iodide staining. Mean percentage values from three independent experiments (*n* = 3) done in duplicate are presented. *p < .05 versus control group.

After treating the MCF-7 and MDA-MB-231 cells with PtPz1–PtPz6 compounds and cisplatin (all in a concentration of 20 μM) for 24 h, the cells were stained with PI, and the cell cycle progression was analysed using a flow cytometer. The results showed that there were significant differences between the PtPz1 and PtPz6 treated groups and the control group with respect to the percentage of cells at the different cell cycle phases (Figure 6 and the supplementary material for publication online). The cells were accumulated considerably at the S and G2/M phases in the PtPz1–PtPz6 treated groups, while the cell population at the G1 phase was significantly reduced. This phenomenon was especially intensified in the case of PtPz1, PtPz2, and PtPz3. When the breast cancer cells were treated with these compounds, the result was a marked accumulation of cells in G2/M phase (2.0 fold) and a reduction in the G1 phase. At the same time, a slight increase in the number of cells in the S phase was observed. All synthesised compounds caused a significant decrease in the percentage of cells in the G1 phase, accompanied by a concomitant percentage increase of cells in the S and G2/M phases. The results indicated a cell cycle arrest at the G1 phase in MCF-7 and MDA-MB-231 cells after PtPz1–PtPz6 compound treatment.

### The effect of PtPz1–PtPz6 on cyclin D1

**Figure 7.**
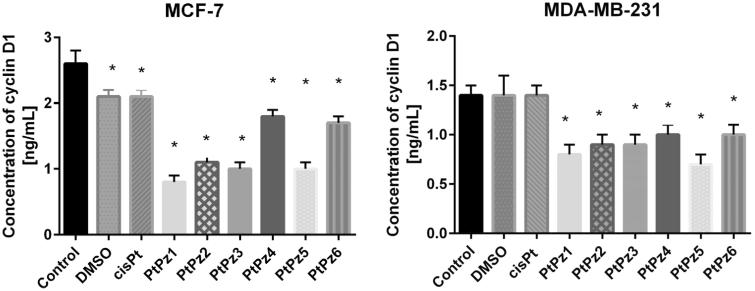
The concentration of human G1/S-specific cyclin D1 in MCF-7 and MDA-MB-231 breast cancer cells after 24 h incubation with PtPz1–PtPz6 (20 μM) and cisplatin (20 μM). *p < .05 versus control group.

The evaluation of cyclin D1 concentration was performed using enzyme-linked immunosorbent assay (ELISA). All novel compounds significantly decreased the concentration of cyclin D1 compared to control value in both tested breast cancer cell lines, but the highest statistically significant decrease was observed after 24 h of incubation with PtPz1 in MCF-7 cells and PtPz5 in MDA-MB-231 cells (Figure 7). The concentration of cyclin D1 after treatment with PtPz1 was 0.8 ng/ml in comparison with untreated MCF-7 cells (2.6 ng/ml). PtPz5 was two times stronger in decreasing the concentration of analysed cyclin than control in MDA-MB-231. The concentration of analysed cyclin was 0.7 ng/ml. The differences were statistically significant (*p* < .05).

### PtPz1–PtPz6 compounds induce apoptosis by DNA fragmentation

**Figure 8.**
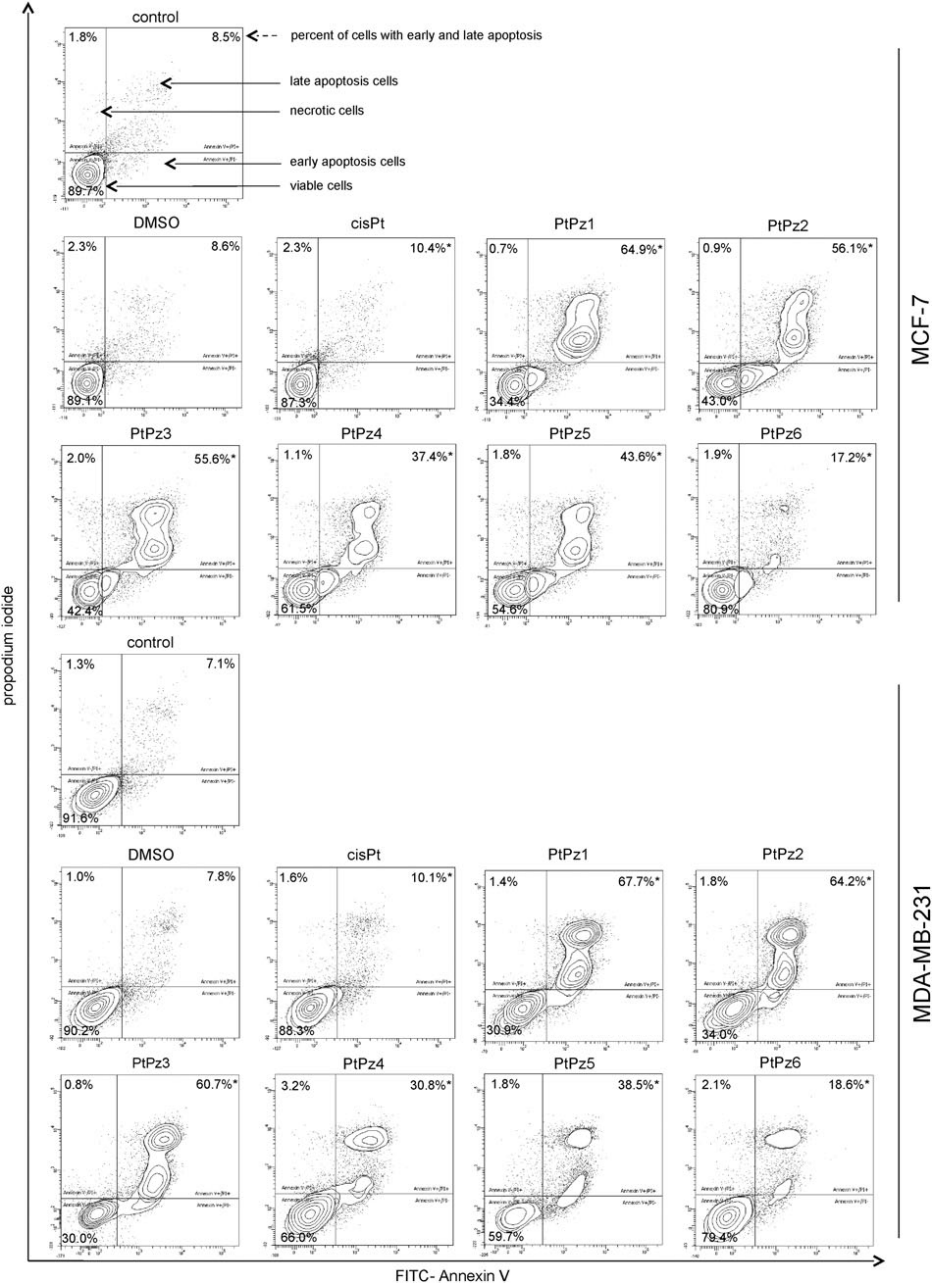
Flow cytometric analysis of MCF-7 and MDA-MB-231 breast cancer cells after incubation with PtPz1–PtPz6 (20 μM) and cisplatin (20 μM) for 24 h and subsequent staining with Annexin V and propidium iodide (PI). Dots with Annexin V−/PI− (left lower square), Annexin V+/PI− (right bottom square), Annexin V+/PI + (right upper square), and Annexin V−/PI+ (left upper square) feature represent intact, early apoptotic, late apoptotic, and necrotic cells, respectively. Mean percentage values from three independent experiments (*n* = 3) done in duplicate are presented. *p < .05 versus control group.

We performed dual Annexin-V and PI staining to assess the apoptosis status of MCF-7 and MDA-MB-231 cells after 24 h of PtPz1–PtPz6 and cisplatin treatment through flow cytometry (Figure 8). Annexin-V can detect the externalisation of phosphatidylserine of the cell membrane, which is one of the hallmarks of apoptosis, whereas PI checks the integrity of the cell membrane^7^. Analysis proved that the tested compounds significantly induced programmed cell death in MCF-7 and MDA-MB-231 cells in comparison with the control (untreated cells), where we detected 89.7% viable cells and 8.5% apoptosis in the case of MCF-7 cells, and 91.6% viable cells and 7.1% apoptosis in MDA-MB-231 cells. The highest pro-apoptotic potential was observed after 24 h of incubation with the compounds PtPz1, PtPz2, and PtPz3 in both breast cancer cells, where we observed 34.4%, 43.0%, and 42.4% viable cells; and 64.9%, 56.1%, and 55.6% apoptotic cells, respectively. The compound with the weakest apoptotic potential was PtPz6, where we detected in both MCF-7 and MDA-MB-231 cell lines 80.9% and 79.4% viable cells; and 17.2% and 18.6% apoptotic cells, respectively. At the same time, it should be noted that in the case of cisplatin, the number of apoptotic cells was only 10.4% in MCF-7 and 10.1% in MDA-MB-231.

**Figure 9.**
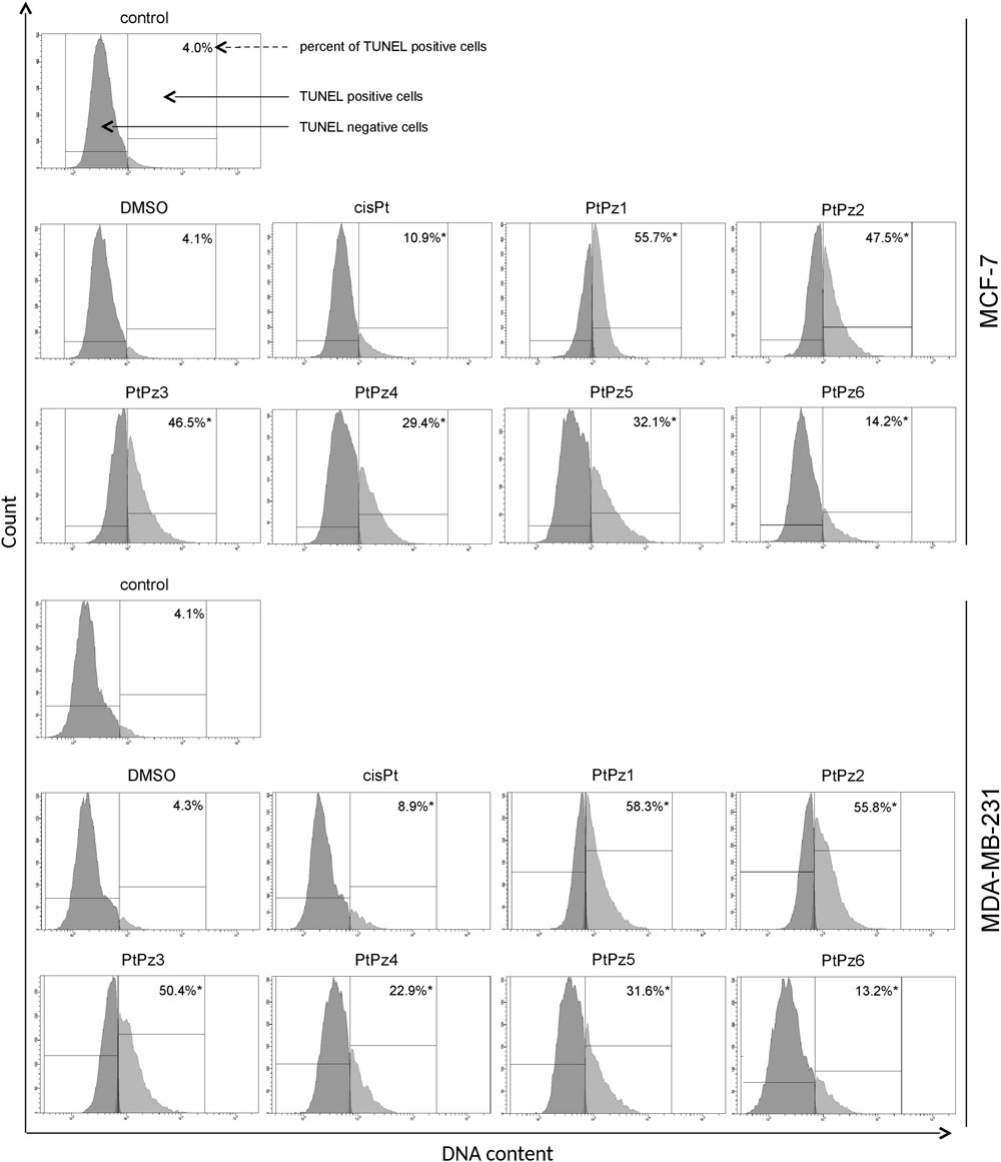
Flow cytometric analysis of DNA fragmentation of MCF-7 and MDA-MB-231 breast cancer cells after 24 h of incubation with PtPz1–PtPz6 (20 µM) and cisplatin (20 µM) using TUNEL assay. Histograms present TUNEL negative and TUNEL positive cells. Mean percentage values from three independent experiments (*n* = 3) done in duplicate are presented. *p < .05 versus control group.

One of the later steps in apoptosis is DNA fragmentation, a process which results from the activation of endonucleases during the apoptotic program. These nucleases degrade the higher-order chromatin structure into fragments of ∼300 kb and subsequently into smaller DNA pieces of about 50 bp in length^9^. Monitoring the pattern of DNA fragmentation by TUNEL assay, which we used in the study, can be a qualitative way to assess apoptosis. MCF and MDA-MB-231 cells were treated with 20 μM of pyrazole-platinum(II) complexes for 24 h and subsequently analysed for DNA fragmentation. According to the different anti-proliferative activities of the tested compounds, we also revealed a certain difference in cellular DNA degradation potency. We found that all the tested compounds were the cause of DNA fragmentation to a greater extent than the control (Figure 9). In the control MCF-7, we detected 4.0% of TUNEL-positive cells. After 24 h of incubation with DMSO and cisplatin, 4.1% and 10.9% of the cells were TUNEL positive. PtPz1–PtPz6 showed a higher percentage with DNA fragmentation compared with cisplatin. The compound PtPz1 had DNA fragmentation at the highest level. In the MDA-MB-231 cells, we observed the highest percentage of TUNEL–positive cells after 24 h of incubation with compounds PtPz1 and PtPz2; 58.3% and 55.8%, respectively. DNA fragmentation for the analysed reference compound was 8.9%. PtPz1–PtPz6 showed a higher percentage with DNA fragmentation compared with cisplatin in both cell lines.

### PtPz1–PtPz6 decrease mitochondrial membrane potential

**Figure 10.**
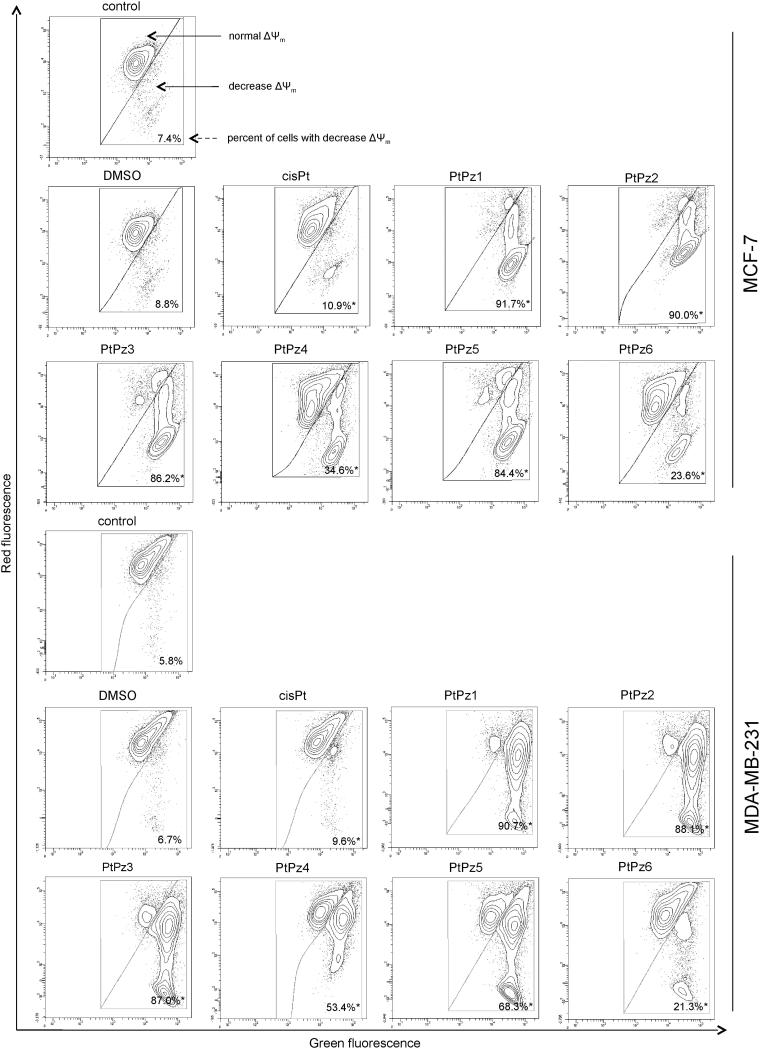
Fluorescence of MCF-7 and MDA-MB-231 breast cancer cells treated for 24 h with PtPz1–PtPz6 (20 μM) and cisplatin (20 μM) incubated with mitochondrial membrane potential probe JC-1. The x- and y-axes are green and red fluorescence, respectively. Mean percentage values from three independent experiments (*n* = 3) done in duplicate are presented. *p < .05 versus control group.

To investigate the cellular mechanism underlying PtPz1–PtPz6 induced intrinsic apoptosis in breast cancer cells, we assessed the alterations of mitochondrial transmembrane potential (ΔΨm) by using lipophilic fluorochrome JC-1 and flow cytometry analysis. In healthy cells, JC-1 penetrates the cell plasma membrane as monomers and is taken up into the mitochondria due to the polarised state of the ΔΨm. The uptake the JC-1 will lead to the aggregation of fluorochromes in the mitochondria. The transition from monomers to aggregates can be detected using a flow cytometer monitoring the red spectral shift. The accumulation of aggregates in the mitochondria is detected at a higher intensity of red fluorescence emission as compared with monomers. Therefore, a loss of ΔΨm will result in increased monomers causing a decrease in the intensity of red fluorescence^9^. As shown in Figure 10, PtPz1–PtPz6 (incubation of 24 h, concentration of 20 μM) in MCF-7 and MDA-MB-231 cells induced an increase in the proportion of cells with depolarised mitochondria. In the case of untreated cells, the decrease in mitochondrial potential was 7.4% in MCF-7 and 5.8% in MDA-MB-231. The compounds that most contributed to the decrease in mitochondrial potential were PtPz1, PtPz2, and PtPz3 (MCF-7: 91.7%, 90.0%, and 86.2%; MDA-MB-231: 90.7%, 88.1%, and 87.0%, respectively). We found that the decrease in MMP caused by PtPz1–PtPz6 was stronger than that evoked by cisplatin. These results are in accord with those obtained in the Annexin-V and PI assay.

### Activation of caspase-3, caspase-8, and caspase-9

**Figure 11.**
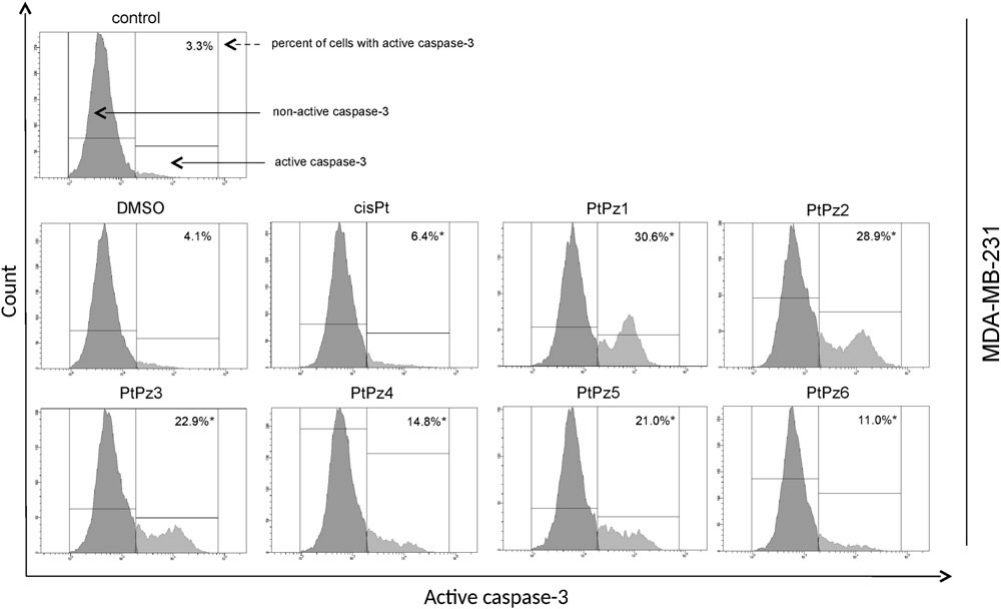
Flow cytometric analysis of populations MDA-MB-231 breast cancer cells treated for 24 h with PtPz1–PtPz6 (20 μM) and cisplatin (20 μM) for active caspase-3. Mean percentage values from three independent experiments (*n* = 3) done in duplicate are presented. *p <.05 versus control group.

To further confirm that PtPz1–PtPz6 compounds induce apoptosis in breast cancer cells MCF-7 and MDA-MB-231, caspase activity (-3, -8 and -9) was assessed. The evaluation was carried out using a flow cytometer. In the case of caspase-3 activity, we found that after 24 h incubation of MDA-MB-231 cells with the compounds in a concentration of 20 μM the cells displayed a significant increase in caspase-3 activity when compared with untreated cells (Figure 11). For the most active compounds (PtPz1 and PtPz2), a nine-fold increase was observed. MCF-7 cells that harbor a spontaneous deletion of 47 bp within exon 3 of the CASP-3 gene lack functional caspase-3^21^.

**Figure 12.**
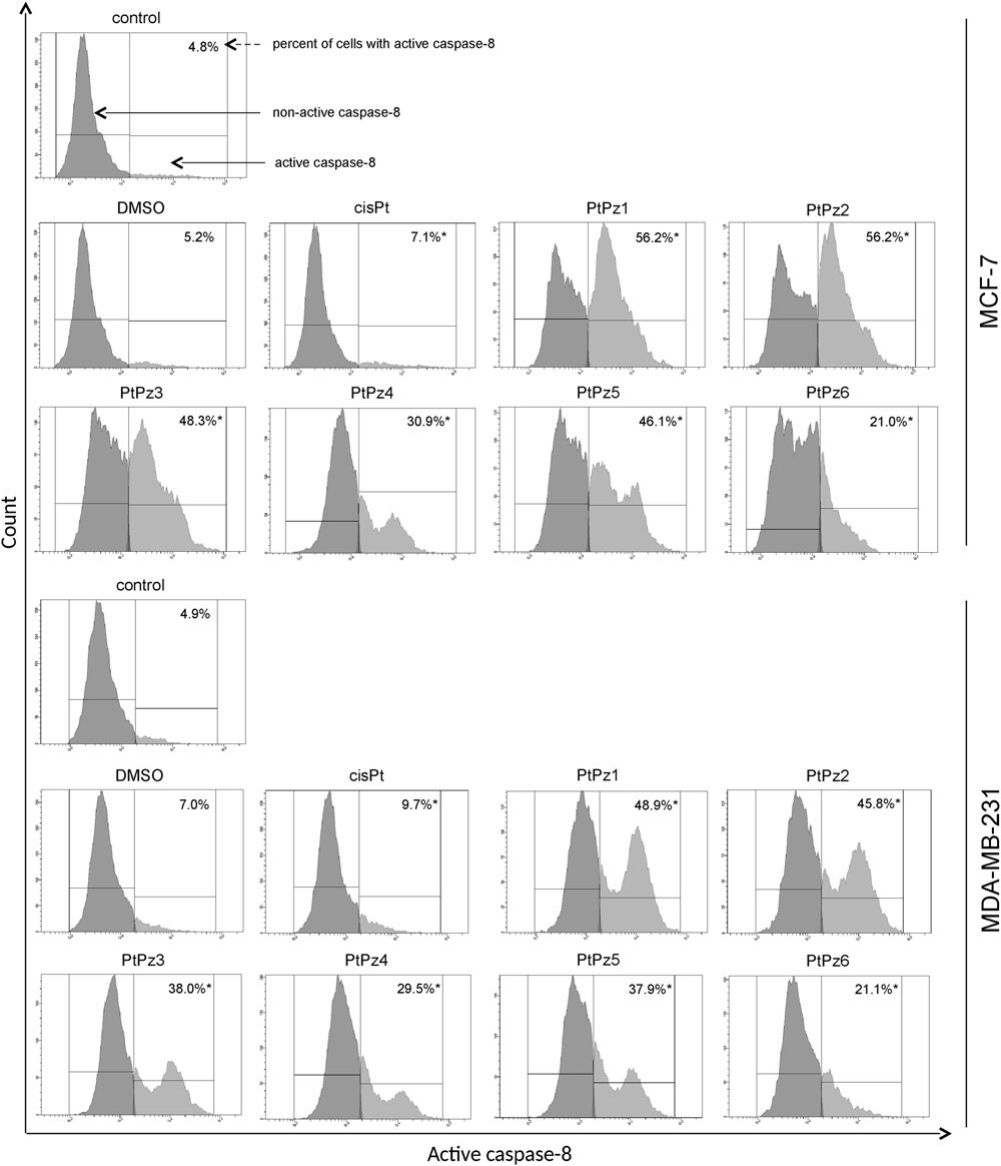
Flow cytometric analysis of populations MCF-7 and MDA-MB-231 breast cancer cells treated for 24 h with PtPz1–PtPz6 (20 μM) and cisplatin (20 μM) for active caspase-8. Mean percentage values from three independent experiments (*n* = 3) done in duplicate are presented. *p <.05 versus control group.

Since caspase-3 is activated in both the intrinsic and extrinsic pathways of apoptosis, caspase-8 activity assays were performed to further understand the role PtPz1–PtPz6 plays in promoting cancerous cell survival. This assay was used to gain further insight into whether novel pyrazole-platinum(II) complexes result in the activation of the caspase-8 mediated extrinsic pathway in both cell lines. It was observed that MCF-7 and MDA-MB-231 cells treated with PtPz1–PtPz6 experienced a significant increase in caspase-8 activity when compared with untreated cells (Figure 12). The highest increase in caspase-8 activity was observed with PtPz1 and PtPz2 compounds, which in MCF-7 cells was almost 12-fold, and in MDA-MB-231 about 10-fold higher compared with the control.

**Figure 13.**
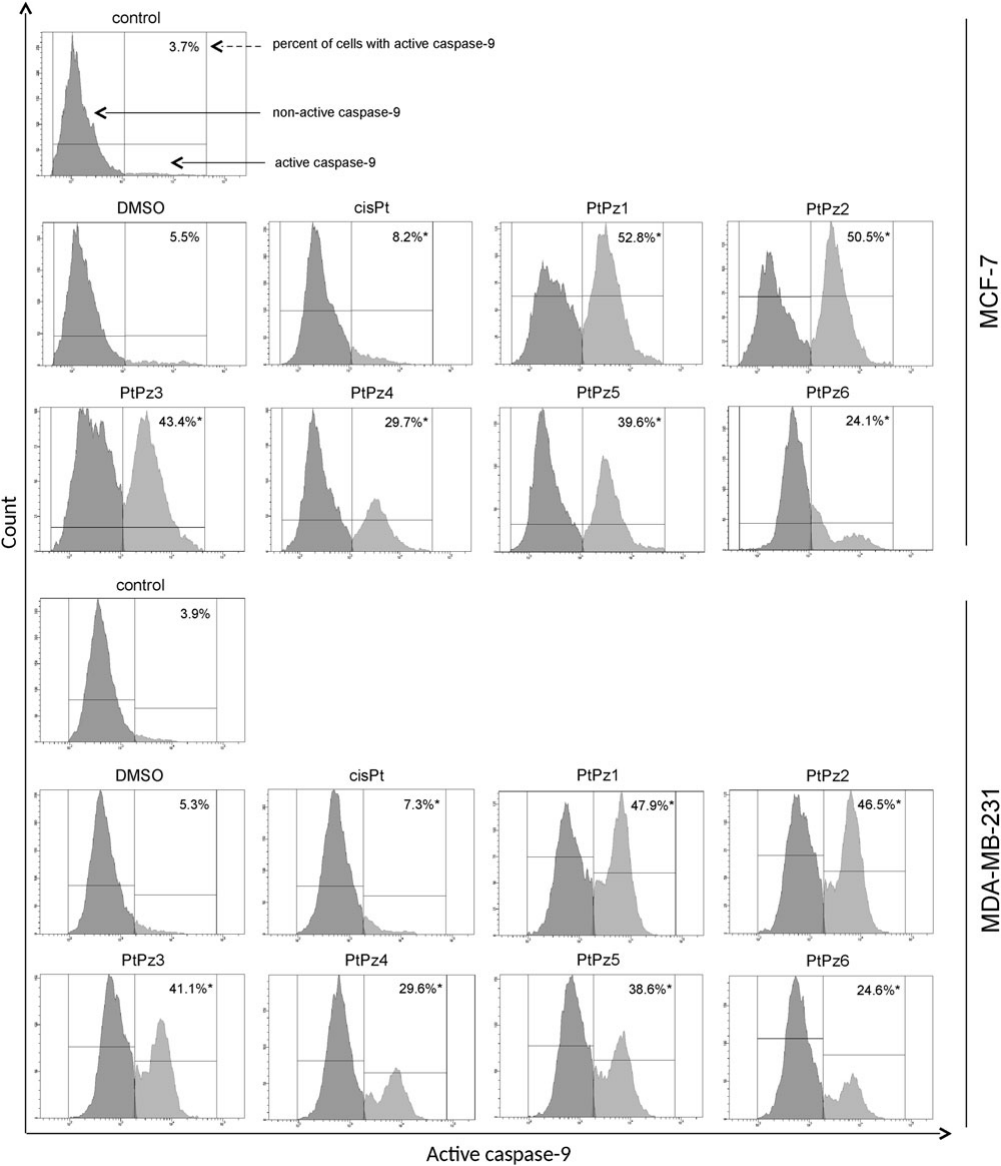
Flow cytometric analysis of populations MCF-7 and MDA-MB-231 breast cancer cells treated for 24 h with PtPz1–PtPz6 (20 µM) and cisplatin (20 µM) for active caspase-9. Mean percentage values from three independent experiments (*n* = 3) done in duplicate are presented. *p < .05 versus control group.

To establish whether breast cancer cells MCF-7 and MDA-MB-231 undergo apoptosis through the caspase-9-mediated intrinsic pathway, caspase-9 activity assays were used. We found that MCF-7 and MDA-MB-231 cells exhibited a significant (10-fold in the case of the most active compounds) increase in caspase-9 activity after treatment with PtPz1–PtPz6 when compared with untreated cells (Figure 13). In the case of the treatment solvent alone, no significant difference was observed in all tested caspase activity. In addition, treatment with cisplatin of both cell lines at 20 μM did not show significant differences in caspase-3, caspase-8, and caspase-9 activity, like in the case of PtPz1–PtPz6.

The results obtained using a flow cytometer were confirmed using the cell imaging method. With the use of immunofluorescence microscopy, we showed increased expression and nuclear translocation of caspase-3, caspase-8, and caspase-9 in cells exposed to PtPz1–PtPz6. Representative photographs are given as supplementary material for publication online.

## Discussion

The basic tasks of modern chemotherapy are the inhibition of tumor cell proliferation. One breast cancer treatment uses alkylating agents, for example platinum(II) complexes. Their main mechanism of action is based on combining with genetic material, which in consequence leads to the retention of replication and transcription processes^22^. In addition, induction of cellular pathways involves the coupling of cisplatin and DNA adenomas formed with caspases, proteins (Bcl-2, p53), and cyclin^23^. All these events lead to the transition of cells into the programmed cell death process^22^^,^^23^. Classic platinum(II) complexes are characterised by a high probability of serious side effects and resistance which results in a lack of therapeutic effect. This fact motivated researchers to look for more efficient, less toxic, and higher-specific platinum(II) complexes^24^.

Of the non-classical platinum compounds, multi-nucleic complexes appear to have the highest potential for high cytotoxicity and maintenance of platinum activity in resistant cell lines. Thanks to the use of ligands within the coordinated platinum zone, compounds with differentiated functions and geometry were obtained^25^. Following this trend, our team synthesised a group of pyrazole-platinum(II) complexes. The electronic properties of pyrazole ligands offer metal centers for late transition metals that are quite electrophilic to allow coordination to biological molecules^26^. Interestingly, pyrazole derivatives are characterised by a wide spectrum of biological activity: anti-tumor, anti-viral, anti-microbial, and anti-inflammatory. The considerable amount of research has reported that pyrazole-based heterocyclic show promising activity against cancer cell lines, including human breast cancer cell lines^26^. The advantage of these derivatives is the ease of complexing with platinum, which may result in an increase in its performance^17^.

It is interesting that the cytotoxicity of cisplatin and pyrazole complexes may considerably depend on the substituents introduced into their pyrazole rings. Sakai et al. showed that while cis-[Pt(Cl_2_)(Pz)_2_] exhibited certain anti-tumor activity, its 3,5-pyrazoledicarboxylic acid analog remained ineffective against human colorectal cell lines DLD-1 and AGS. They suspected that the introduction of the carboxyl group might result in poor electron density at pyrazole moiety and also generate larger steric hindrances^27^.

Additionally, Ciesielska et al. showed that the N-hydroxymethyl-3,5-dimethylpyrazole substituent in the complexes cisplatin with pyrazole causes much more activity than the pyrazole itself in the case of L1210 murine leukemia cells^28^. The results of our experiment may be an extension of the phenomenon observed by the above-mentioned authors. Our study was concerned with the effects of novel pyrazole-platinum(II) complexes (PtPz1–PtPz6) on breast cancer cells: estrogen receptor-positive and estrogen receptor-negative. Our research showed that PtPz1–PtPz6 have a higher cytotoxicity in comparison with cisplatin in relation to MCF-7 and MDA-MB-231 cells. Breast cancer cells treated with compounds showed a reduction in cell viability in a dose-dependent manner. The highest cytotoxicity was exhibited by: PtPz1 - [Pt_2_N-hydroksymethyl-3,5-dimethylpyrazole_4_(berenil)_2_]Cl_4_; PtPz2 - [Pt_2_3,5-dimethylpyrazole_4_(berenil)_2_]Cl_4_; and PtPz3 - [Pt_2_3,4-dimethylpyrazole_4_(berenil)_2_]Cl_4_.

We should note that the pyrazole itself as a substituent ([Pt_2_pyrazole_4_(berenil)_2_]Cl_4_) showed much lower activity than the above-mentioned compounds, both in the MCF-7 and MDA-MB-231 cell lines. At the same time, the tested compounds showed lower cytotoxicity on human skin fibroblasts than on tumor cells. The cytotoxic activity of these compounds with different substituents on the pyrazole ring increased in the following order: PtPz1 > PtPz2 > PtPz3 > PtPz5 > PtPz4 > PtPz6. Compounds PtPz1, PtPz2, and PtPz3 with methyl substituents at the pyrazole ring showed stronger activity than the pyrazole or ethylpyrazole containing complexes. It is believed that the type of pyrazole ligand appears to affect activity. For example, the activity of complex 4-pyrazolyl-1,8-naphthalimide is significantly higher than that of 1,8-naphthalimide^29^.

Previous studies also show that the pyrazol[1,5-*a*][1,3,5]triazines are more active than 1,3,5-triazine^30^. Additionally, it is believed that the bulkiness of the pyrazole ligands also appears to affect activity. For example, the activity of dichloro-bis(pyrazole)platinum(II) was significantly higher than that of cisplatin^26^. Gumus et al. showed that the bulky dichloro-2–(2-pyridyl)benzimidazoleplatinum(II) was more active than cisplatin. The steric hindrance due to 2–(2-pyridyl)benzimidazole ligands was found to reduce rapid detoxification by thiol containing molecules, as compared with the ammonia ligands in cisplatin. There is a probability that such bulky ligands prevent translabilisation and undesired displacement of the non-leaving ligand by other nitrogen donors^31^. Hence, it is possible that complexes PtPz1–PtPz6 could be acting through a similar mechanism that induces the formation of DNA-adducts, promoting the activation of apoptosis. Disruption of cell cycle progression is regarded as an important strategy for eliminating cancer cells. Many anticancer compounds exert their growth inhibitory effect either by arresting the cell or by inducing apoptosis or a combined effect of both cycle arrest and apoptosis^32^. Moreover, regulation of the cell cycle and apoptosis are considered to be effective approaches in the development of cancer therapeutics^33^. There are many reports that the pyrazole derivatives exhibited cell cycle arrest properties. Qin et al. showed that compounds with potential anti-neoplastic properties containing the pyrazoline ring in their structure caused an accumulation of cells in the G2/M phase of the cell cycle (in the case of human cancer cell lines: MIAPaCa-2, MCF-7, and HeLa)^34^. Moreover, Kamal et al. showed on the example of cell lines A549 and IMR32 that compounds containing pyrazole rings in their structure caused a G1 phase delay/arrest and increased the G2/M phase^35^.

Cisplatin is a known anti-tumor drug, but its mechanisms of action have not been fully elucidated. The cell cycle analysis done by Velma et al. in HL-60 cells indicated that, at lower doses of cisplatin treatment, the cells were arrested at sub-G1, S, or G2 checkpoints; and as the cisplatin dose and treatment time increased, the cells accumulated more in the sub-G1 phase^36^. In current study, we observed significant deregulation of the cell cycle in both cell lines after treatment with PtPz1–PtPz6. Flow cytometry analysis demonstrated clear decrease the number of cells in the G1 phase. At the same time, there was an increase in the number of cells in the S and G2/M phases. The cell cycle disturbances were associated with an inhibition of cell proliferation, which is in agreement with results reported by Kumar et al. and Wang et al. in their cell cycle disturbance studies^37^^,^^38^. An increase in the cell cycle of the G2/M phase may indicate that DNA strands have been damaged, which is compatible with one of the mechanisms of cisplatin^23^. During this phase, tumor cells may attempt to repair the DNA, leading to the activation of the apoptosis pathway. In addition, the present study demonstrated that cyclin D1, which is a key protein in cell cycle progression^39^ was downregulated in PtPz1–PtPz6 treated cells compared with untreated cells. It indicates that cyclin D1 may be associated with inhibit cell growth.

Apoptosis is a complex process that can be triggered by many different factors. A major role in this process is played by the mitochondria, whose role in apoptosis has not been fully explained. There are two concepts of perceiving mitochondria in the apoptosis process. One of them suggests that mitochondria play a key role in apoptosis by releasing cytochrome c and other proteins that play an essential role in the activation of procaspase-9. This concept also assumes that caspase-9 activates caspase-3. Caspase-3 is often referred to as the “killer” in the apoptosis process because it cleaves many proteins leading to the loss of normal cellular structure, resulting in cell death. Hence, this assumption suggests that mitochondria are essential elements in the process of apoptosis. Another concept assumes that mitochondria act as facilitators rather than the basic elements of programmed cell death. For example, some signals may lead to caspase activation without prior inclusion in the mitochondria. Such a model assumes that caspases are the primary factors causing cell death.

Given the complexity of the apoptosis process, there are probably many mechanisms that lead to this process^40^. Because earlier research showed that pyridine-platinum(II) complexes induced apoptosis via the mitochondria pathway and the receptor pathway in Ishikawa endometrial cancer cells and breast cancer cells^8^^,^^9^, we hypothesised that PtPz1–PtPz6 may exert apoptosis through a similar pathway. To confirm this, we showed that PtPz1–PtPz6 were able to induce apoptosis using flow cytometric analysis. Furthermore, increased exposure to PtPz1–PtPz6 led to a significant decrease in mitochondrial transmembrane potential, confirming that apoptosis was triggered through the mitochondrial pathway.

Reduction of MMP is known as an early apoptotic event and associated with the activation of caspases^13^. Our study showed that PtPz1–PtPz6 in MDA-MB-231 cells were able to activate caspase-8 and -9 followed by downstream caspase-3. A similar situation was observed for MCF-7 cells, except for caspase-3, which was not detected in this cell line. Despite the fact that MCF-7 lack the expression of executor caspase-3, known as CPP32, they undergo apoptosis after treatment with anti-cancer agents. The studied cell line has confirmed 47 kb deletion in exone 3 of the CPP32 gene. However, it appears that not only the activation of caspase-3, but also caspase-7, by the action of initiator caspase-8 and -9, enhanced the apoptosis process. Caspase-7 is strongly associated with caspase-3 and they exhibit the same substrate specificity *in vitro*^41^. Our results suggest that pyrazole-platinum(II) complexes regulate the apoptosis of breast cancer cells independent of caspase-3 status.

Pinato et al. previously demonstrated that the pro-apoptotic activity of platinum compounds is associated with the production of DNA strand breaks^42^. Moreover, it is believed that pyrazole moiety damages the DNA strand, affecting the regulation of gene expression and epigenetic signaling pathways in tumors and numerous other diseases^43^. PtPz1–PtPz6 produced more extensive DNA fragmentation than cisplatin. This degradation represents the double strand breaks when the DNA helix existed in a double stranded form. Previous studies showed that pyrazoles have anti-tumor activity on different types of cancer cells: non-small cell lung cancer, human hormone-independent prostate cancer, stomach cancer, myeloid leukemia, sarcoma, gastric cancer, and cervical carcinoma^44^. Therefore, it is possible that pyrazole-platinum(II) complexes may be an effective scaffold for further research on complexes with potential anti-tumor activity.

## Conclusion

It is known that apoptosis contributes to the cytotoxic effects of anti-cancer drugs. For this reason, compounds with pro-apoptotic activity should be considered promising candidates for anti-cancer drug development. Our study demonstrated that the pyrazole-platinum(II) complexes can modulate both the apoptotic pathways and activate initiator as well executioner caspases. Moreover, although the structure–activity relationship observed on the reaction level of the tested pyrazole platinum complexes *in vitro* does not entirely explain the mechanism of their cytotoxic action, our results suggest that the presence of the methyl substituent at the N3 position of the pyrazole ring is an advantageous feature for the pyrazole platinum complex’s DNA crosslinking activity. Therefore, further understanding of how different substituted pyrazole-platinum complexes modify DNA, and how these modifications are processed in the cells should provide a rational basis for the design of new pyrazole-platinum drugs.

## Supplementary Material

IENZ_1471687_Supplementary_Material.pdf
