# Supplementary material for: A novel series of pyrazole-platinum(II) complexes as potential anti-cancer agents that induce cell cycle arrest and apoptosis in breast cancer cells
Source: J Enzyme Inhib Med Chem. 2018 Jun 4;33(1):1006–23. doi: 10.1080/14756366.2018.1471687 (PMC6009892; doi:10.1080/14756366.2018.1471687)
Supplement: IENZ_1471687_Supplementary_Material.pdf [file IENZ_A_1471687_SM4369.pdf]

Table S1

	cisPt	PtPz1	PtPz2	PtPz3	PtPz4	PtPz5	PtPz6
MCF-7 (24 h incubation)	>50 μM	17±2 μM	20±2 μM	23±1 μM	32±2 μM	28±2 μM	>50 μM
MCF-7 (48 h incubation)	>50 μM	11±1 μM	13±1 μM	18±2 μM	29±2 μM	22±2 μM	48±2 μM
MDA-MB-231 (24 h incubation)	>50 μM	16±1 μM	17±2 μM	20±2 μM	33±3 μM	29±1 μM	>50 μM
MDA-MB-231 (48 h incubation)	>50 μM	10±1 μM	11±1 μM	16±1 μM	29±2 μM	19±2 μM	39±2 μM
Fibroblasts (24 h incubation)	>50 μM	24±3 μM	27±2 μM	32±1 μM	>50 μM	35±1 μM	>50 μM
Fibroblasts (48 h incubation)	>50 μM	19±2 μM	24±2 μM	28±2 μM	46±2 μM	31±2 μM	>50 μM

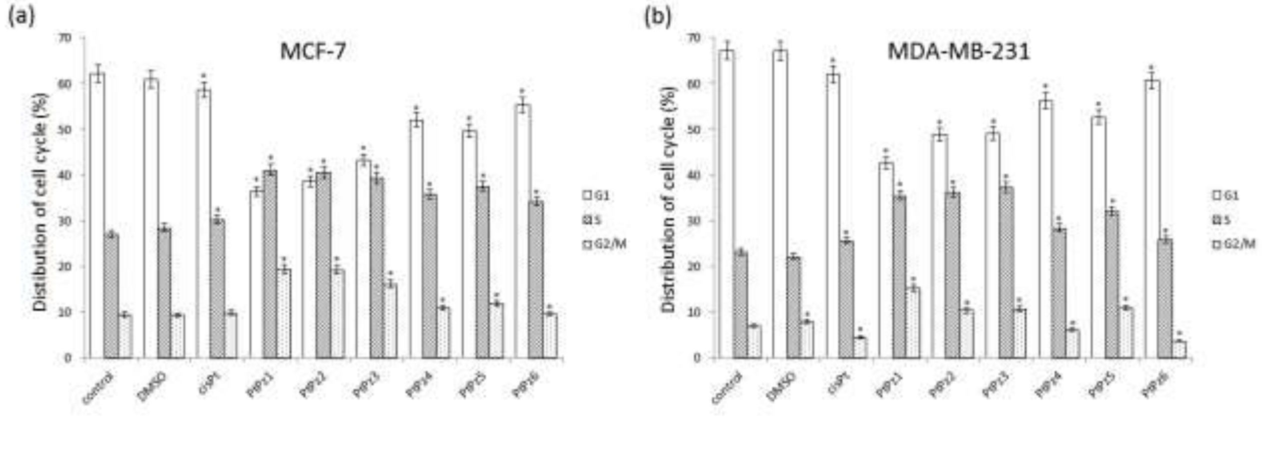

Figure S1

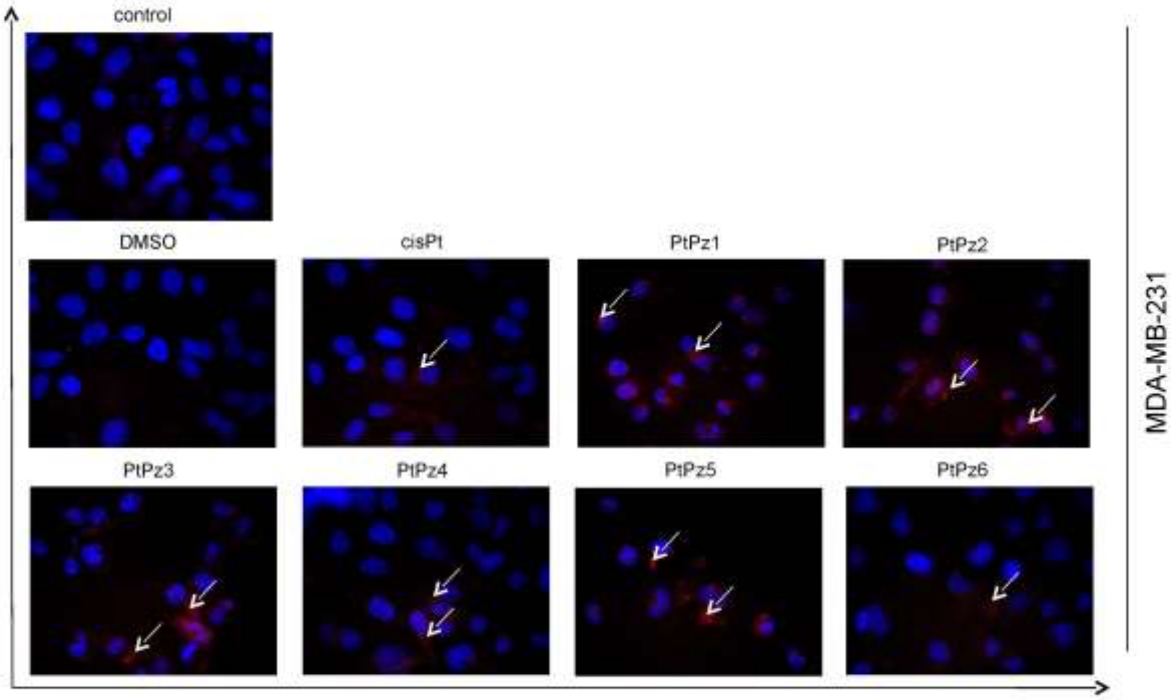
control
DMSO
cisPt
PtPz1
PtPz2
PtPz3
PtPz4
PtPz5
PtPz6
MDA-MB-231

Figure S2

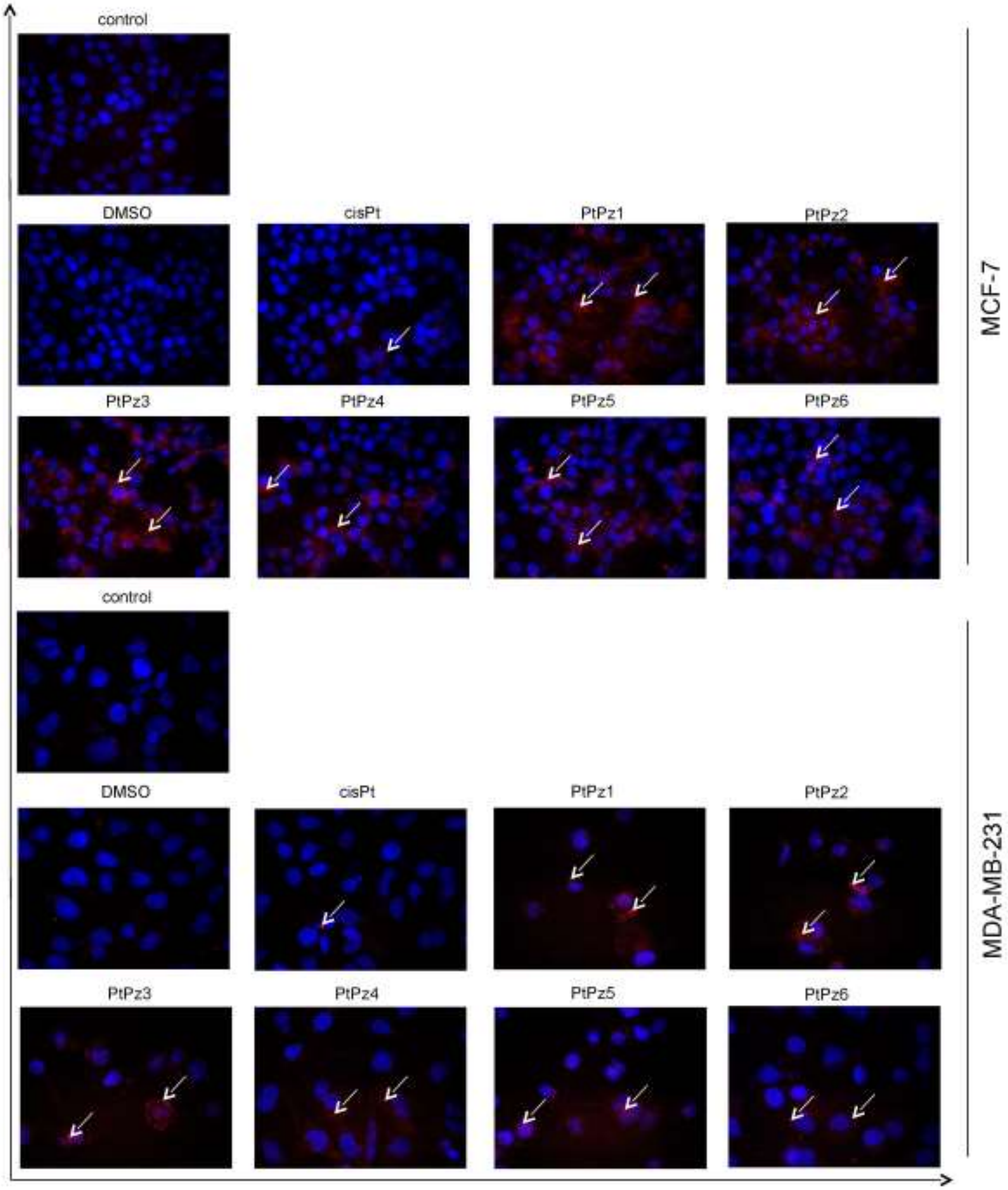
control
DMSO
cisPt
PtPz1
PtPz2
PtPz3
PtPz4
PtPz5
PtPz6
MCF-7
control
DMSO
cisPt
PtPz1
PtPz2
PtPz3
PtPz4
PtPz5
PtPz6
MDA-MB-231

Figure S3

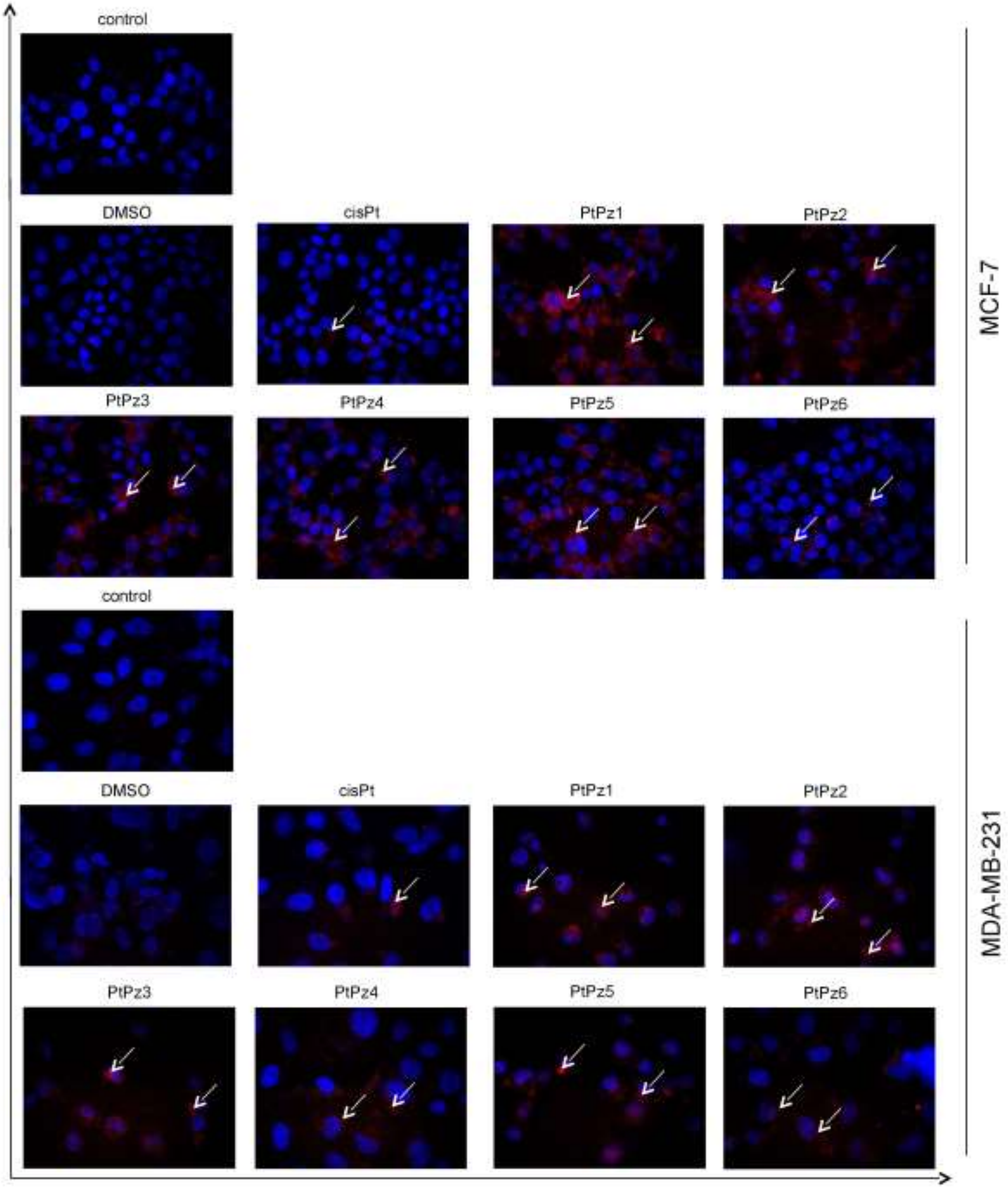
control
DMSO
cisPt
PtPz1
PtPz2
PtPz3
PtPz4
PtPz5
PtPz6
MCF-7
control
DMSO
cisPt
PtPz1
PtPz2
PtPz3
PtPz4
PtPz5
PtPz6
MDA-MB-231

Figure S4
